# Erector spinae plane block reduces postoperative nausea and vomiting: a systematic review and meta-analysis of 44 randomized trials

**DOI:** 10.3389/fmed.2025.1749998

**Published:** 2026-01-16

**Authors:** Chong Zhao, Minmin Zhu, Jinjin Jian, Jinfang Zeng

**Affiliations:** 1 Department of Anesthesiology, Sir Run Run Shaw Hospital, Zhejiang University School of Medicine, Hangzhou, China; 2 Department of Anesthesiology, Jiangnan University Medical Center (Wuxi No. 2 People’s Hospital, Affiliated Wuxi Clinical College of Nantong University), Wuxi, China; 3 Department of Anesthesiology, Affiliated Hospital of Jiangnan University, Wuxi, China

**Keywords:** erector spinae plane block, ESPB, meta-analysis, nausea, vomiting

## Abstract

**Background:**

Postoperative nausea and vomiting (PONV) is a prevalent complication and remains a significant clinical challenge. The erector spinae plane (ESPB) block has been shown to offer significant pain relief during and after surgical procedures, positioning it as a potentially beneficial anesthetic technique. However, limited evidence currently supports its effectiveness in reducing nausea and vomiting specifically. This meta-analysis aims to examine the impact of ESPB on PONV rates, assessing whether the block offers measurable benefits for this common postoperative issue, in addition to its recognized analgesic effects.

**Methods:**

Two researchers conducted a comprehensive search across three databases—PubMed, Embase, and the Cochrane Central Register of Controlled Trials—using keywords such as “erector spinae plane block, meta-analysis, nausea, and vomiting” to identify all relevant literature. The data obtained from these studies were then analyzed through meta-analysis, utilizing Review Manager software to synthesize findings and assess overall outcomes.

**Results:**

In this meta-analysis, 44 trials involving 2,830 patients were analyzed. The ESPB was found to significantly decrease the incidence of nausea (risk difference (RD) = -0.16, 95% confidence interval (CI): -0.21—0.12) and vomiting (RD = -0.12, 95% CI: -0.17—0.07) compared to no ESPB. Additionally, ESPB decreased the dosage of morphine [standardized mean difference (SMD) = -0.86, 95% CI: -1.54 to -0.18], fentanyl (SMD = -2.96, 95% CI: -5.13 to -0.79), and tramadol (SMD = -1.43, 95% CI: -2.32 to -0.55) when compared to no ESPB. It also reduced VAS movement at 24 h (SMD = -1.58, 95% CI: -3.04 to -0.13) and lowered the occurrence of dizziness (RR = 0.43, 95% CI: 0.18–1.02), while prolonging the likelihood of itching (RR = 0.39, 95% CI: 0.25–0.61). We assessed the outcomes of nausea and vomiting with GRADE, and they were high to moderately certain, respectively. In addition, our analysis of trial sequences found adequate sample sizes for reductions in the incidence of nausea and vomiting.

**Conclusion:**

Our meta-analysis found that ESPB may be able to reduce the incidence of nausea and vomiting and improve the quality of patients’ postoperative recovery and trial sequential analysis confirmed an adequate sample size for this conclusion.

**Systematic review registration:**

https://www.crd.york.ac.uk/PROSPERO/, identifier CRD42024604805.

## 1 Introduction

PONV is a common complication in the perioperative period, especially among non-smokers, women, and those with a history of motion sickness. These symptoms can result in dehydration and electrolyte imbalances, and in severe cases, they may lead to aspiration, putting the patient’s life at risk. These complications can adversely affect patient outcomes, extend hospital stays, and complicate the overall recovery process (1). These symptoms can also increase postoperative pain due to abdominal muscle strain, delay wound healing, and extend hospital stays, affecting overall recovery (2). Nausea and vomiting cause patients to feel anxious and uneasy (3). Several factors influence the occurrence of nausea and vomiting, such as the patient’s age and gender, smoking habits, previous experiences with motion sickness, the specific surgical procedure performed, and the anesthetic agents utilized (4). Additionally, individual sensitivity, postoperative pain management, and psychological factors like anxiety also play significant roles (5). Recognizing these influences is essential for developing effective prevention and treatment strategies.

As its name implies, the ESPB is an analgesic technique involving the injection of a local anesthetic near the erector spinae muscle to alleviate pain (6). The primary target of the block is the dorsal ramus of the spinal nerve, with the main analgesic effects observed primarily in the thoracic and abdominal regions. Research has demonstrated that ESPB can markedly decrease opioid consumption while enhancing patient comfort, which is especially advantageous for patients undergoing thoracic and abdominal surgeries (7). Advancements in ultrasound technology have enhanced the precision of nerve blocks, significantly improving their accuracy in surgical procedures, particularly for thoracic and abdominal surgeries. As a result, ESPB is gaining recognition as a valuable tool in modern anesthesia practice (8). It has been associated with lower rates of postoperative nausea and vomiting, making it a favorable option for enhancing recovery in surgical patients (9). Some studies have suggested that ESPB may have a beneficial effect on reducing PONV by modulating the afferent pain signals and potentially reducing the need for systemic opioids, which are known to be associated with PONV (10, 11). However, the current literature remains inconclusive, and there is a need for a thorough analysis of the existing evidence.

This meta-analysis aims to assess the effectiveness of ESPB in preventing PONV by systematically reviewing relevant randomized controlled trials.

## 2 Methods

A thorough meta-analysis was conducted to evaluate the impact of the ESPB on PONV, following PRISMA guidelines. Registered in PROSPERO (CRD42024604805), this study does not require ethical consent. PONV was used as a proxy for postoperative nausea when studies did not report nausea separately. When both composite PONV and separate nausea and/or vomiting data were available, we preferentially extracted and analyzed the separate counts for nausea and vomiting; composite PONV data were not artificially disaggregated. For the vomiting outcome, only trials that explicitly reported vomiting events were included. Nausea severity was assessed using a grading scale, where a score of 0 indicated no nausea, 1 represented mild nausea, 2 signified moderate nausea, and 3 indicated severe nausea. To harmonize differences in postoperative assessment timing, we adopted a standard 0–24 h postoperative window. When studies reported multiple time points, we preferentially used the 24 h value or the cumulative incidence over 0–24 h. If only shorter or longer intervals were reported, we used the interval whose mid-point was closest to 24 h (for example, 0–12 h or 0–48 h), to maximize comparability across studies. This systematic classification allows for a clearer understanding of the patient’s experience and the overall impact of the treatment. Detailed PONV definitions, assessment tools, and assessment time points for each included trial are summarized in Supplementary Table 6.

### 2.1 Search approach and eligibility standards

Z.J.F. and J.J.J. systematically searched the Cochrane Library, Embase, and PubMed for studies related to paravertebral block or ESPB, as well as nausea, vomiting, PONV, and surgical, anesthetic, or postoperative care factors. This investigation continued until July 13, 2024, without language restrictions. Additionally, they conducted a comprehensive review of the bibliographies from initial reports, critiques, and case studies to confirm their findings.

### 2.2 Research selection

Z.M.M. and J.J.J. independently screened the articles and extracted data on nausea and vomiting incidence, total sample size, type of surgery, duration of anesthesia and surgery, anesthetic regimen, local anesthetic concentration and dose, and year of publication. Any disagreements were resolved collaboratively through discussion among all authors to reach a consensus.

#### 2.2.1 Inclusion criteria

Studies were included if they met all of the following eligibility criteria: (1) Study population consisting of patients undergoing surgery with general anesthesia; (2) Intervention involving ESPB. (3) Comparator: placebo versus no treatment, comparative study on the exclusion of ESPB versus other types of nerve blocks; (4) Primary outcomes: frequency of nausea or vomiting; secondary outcomes: VAS, opioid dosages, probability of dizziness and pruritus; (5) Types of studies: randomized controlled trials (RCTs).

#### 2.2.2 Exclusion criteria

(1) Solely the registration number or abstract; (2) Absence of data; (3) Incorrect statistical evaluation; (4) Comparing ESPB with other nerve blocks.

### 2.3 Information extraction and evaluation of bias risk

A pair of researchers (Z.C. and J.J.J.) separately evaluated the quality of the study employing the Cochrane Collaboration’s bias risk instrument. Our assessment encompassed six distinct areas: selective reporting, partial result data, various biases, generation of random sequences, concealment of allocation, and the use of blind methods. Each category is categorized into high risk, low risk, or ambiguous risk. In addition, we extracted key ESPB technical characteristics, including block level, timing, and whether sensory testing or block verification was reported; these data are summarized in Table 1.

**TABLE 1:** General information of patients with incidence of postoperative nausea and vomiting.

Author (year)	Age	Sex(M/F)	Type of surgery	Surgical category	Anesthetic type	ESPB level	Sensory testing/block verification	Comparisons (Group)	Time of administration	Nausea	Vomiting	Total
Abdelgalil, A. S. 2022 (53)	18–65 years	12/18	Open nephrectomy for renal malignancies	Urologic	General anesthesia (opioid-free TIVA vs. balanced GA with opioids)	Unilateral continuous (T8–T9)	Absence of pinprick sensation at the block’s dermatomal location 30 min after injection	ESPB Group 0.25% bupivacaine 50 mg	Preoperative	5	3	30
		21/9						Control Group 0.9% saline 20 mL		11	8	30
Abu Elyazed, M. M. 2019 (52)	18–65 years	7/23	Open epigastric hernia repair	Abdominal/ abdominal wall	General anesthesia (sevoflurane inhalation + remifentanil)	Bilateral (T7)	Not mentioned	ESPB Group 0.25% bupivacaine 50 mg	Preoperative	2	–	30
		9/21						Control Group 0.9% saline 20 mL		3	–	30
Avis, G. 2022 (51)	> 18 years	19/6	Lumbar spine surgery	Spine	General anesthesia (balanced anesthesia with opioids)	Bilateral (L3)	Not mentioned	ESPB Group 0.375% ropivacaine 150 mg	Preoperative	0	–	25
		18/6						Control Group 0.9% saline 40 mL		2	–	25
Bryniarski, P. 2021 (50)	18–70 years	15/19	Percutaneous nephrolithotomy	Urologic	General anesthesia (sevoflurane + remifentanil)	Unilateral (T7–T8)	Not mentioned	ESPB Group 0.5% bupivacaine 100 mg	Before general anesthesia induction	6	–	34
		22/12						Control Group 0.9% saline 20 mL		5	–	34
Canıtez, A. 2021 (49)	18–65 years		Laparoscopic cholecystectomy	Abdominal/ hepatobiliary	General anesthesia (sevoflurane)	Bilateral (T7–T8)	Not mentioned	ESPB Group 0.5% bupivacaine 100 mg	At the end of the surgery and before tracheal extubation	7	3	41
								Control Group 0.9% saline 20 mL		16	8	41
Chiraya, S. 2023 (48)	18–65 years		Thoracolumbar spine surgery	Spine	General anesthesia (desflurane + N2O/O2)	Bilateral (T9–T10)	Not mentioned (block performed after general anesthesia induction, unable to assess dermatomal block)	ESPB Group 0.25% bupivacaine 50 mg	After induction of anesthesia	8	–	35
								Control Group 0.9% saline 20 mL		14	–	35
Ciftci, B. 2020 (47)	18–65 years	16/14	Video-assisted thoracic surgery	Thoracic/cardiac	General anesthesia (desflurane + remifentanil infusion)	Unilateral (T5–T6)	Not mentioned	ESPB Group 0.25% bupivacaine 50 mg	Before general anesthesia induction	5	5	30
		15/15						Control Group 0.9% saline 20 mL		22	9	30
Domagalska, M. 2024 (46)	10–18 years	15/15	Pediatric idiopathic scoliosis surgery	Spine	General anesthesia (sevoflurane balanced GA)	Bilateral bilevel (T4–T8)	Not mentioned	ESPB group 0.2% ropivacaine 20 mg	After the induction of general anesthesia	0	–	30
		14/16						Control Group 0.9% saline 10 mL		14	–	30
Dubilet, M. 2023 (45)	> 18 years	42/8	Open oncologic abdominal surgery	Abdominal/ abdominal wall	General anesthesia (TIVA propofol + remifentanil)	Bilateral (T7–T9)	Not mentioned	ESP group 0.2% ropivacaine60 mg	Preoperative	11	–	50
		38/12						Control Group 0.9% saline 30 mL		27	–	50
Elshafie, M. A. 2022 (44)	18–65 years	10/10	Cirrhotic patients undergoing hepatic resection	Abdominal/ abdominal wall	General anesthesia (TIVA propofol + remifentanil TCI)	Bilateral (T4)	Not mentioned	ESPB Group 0.25% bupivacaine 50 mg	After induction of anesthesia	0	–	20
		7/13						Control Group 0.9% saline 20 mL		10	–	20
Fu, J. 2020 (43)	20–65 years	16/14	Hepatectomy	Abdominal/ abdominal wall	General anesthesia (sevoflurane + fentanyl)	Bilateral (L3–L4)	Not mentioned	ESPB Group 20 mL 0.5% ropivacaine100 mg	Preoperative	2	–	30
		17/13						Control Group 0.9% saline 20 mL		8	–	30
Gado, A. A. 2022 (42)	6 months –7 years	22/28	Lumbar spinal fusion surgery	Spine	General anesthesia (sevoflurane balanced)	Bilateral (T4–T5)	Not mentioned	ES group 0.25% bupivacaine 12.5 mg	After induction of anesthesia	–	2	50
		21/27						Control Group 0.9% saline 6 mL		–	4	48
Gişi, G. 2023 (41)	–	13/8	Cholecystectomy	Abdominal/ hepatobiliary	General anesthesia (TIVA propofol + remifentanil)	Bilateral (L3–L4)	Not mentioned	ESPB Group 0.25% bupivacaine 50 mg	Preoperative	4	–	21
		8/13						Control Group 0.9% saline 20 mL		9	–	21
Gökduman, H. C. 2024 (40)	> 65 years	24/38	Breast surgery	Breast	Local anesthesia (awake outpatient ESPB only)	Unilateral (T7)	Not mentioned	ESPB Group 0.5% bupivacaine 100 mg	In the recovery room	3	–	62
		18/30						Control Group 0.9% saline 20 mL		9	–	46
Gürkan, Y. 2018 (39)	18–65 years		Hepatectomy	Abdominal/ abdominal wall	General anesthesia (balanced GA with opioids)	Unilateral (T4)	Not mentioned	ESPB Group 0.25% bupivacaine 50 mg	Before induction of anesthesia	8	3	25
								Control Group 0.9% saline 20 mL		10	4	25
Hacıbeyoglu, G. 2022 (38)	18–65 years	19/6	Lumbar spine surgery	Spine	General anesthesia (TIVA propofol + remifentanil)	Bilateral (T8)	Loss of hot-cold sensation above and below bilateral T8 dermatome 20 min after block	ESPB group 0.375% bupivacaine 75 mg	Before induction of anesthesia	19	–	25
		19/6						Control Group 0.9% saline 20 mL		25	–	25
Hamdi, A. A. 2023 (37)	20–65 years		Minimally invasive mitral valve surgery	Thoracic/cardiac	General anesthesia (sevoflurane + remifentanil)	Bilateral (L3–L4)	Not mentioned	ESPB Group 0.25% bupivacaine 50 mg	Preoperative	2	–	22
								Control Group 0.9% saline 20 mL		5	–	22
Hoogma, D. F. 2023 (36)	18–80 years		Cesarean section	Obstetric	General anesthesia (sevoflurane balanced)	Unilateral (T5)	Sensory block evaluated via ether swab and pinprick at midaxillary line (surgical and non-surgical sides) at 2 and 18 h post-extubation	ESPB Group 0.5% ropivacaine 150 mg	After completion of surgery	14	–	36
								Control Group 0.9% saline 30 mL		18	–	36
Hu, J. 2022 (35)	-	0/30	Gastrectomy	Abdominal/ abdominal wall	General anesthesia (sevoflurane + remifentanil)	Bilateral (T9–T10)	Not mentioned	ESPB Group 0.25% ropivacaine 50 mg	Preoperative	3	–	30
		0/30						Control Group 0.9% saline 20 mL		7	–	30
Jeong, H. 2022 (34)	20–70 years	20/8	Lumbar laminoplasty	Spine	General anesthesia (balanced GA)	Bilateral (T5)	Decrease in pinprick sensation at the back and axillary line 15 min after block	ESPB Group 0.375% ropivacaine 112.5 mg	Before induction of anesthesia	7	–	28
		17/13						Control Group 0.9% saline 30 mL		4	–	30
Jin, Y. 2021 (33)	> 18 years		Posterior lumbar interbody fusion	Spine	General anesthesia (TIVA propofol + remifentanil)	Bilateral (T7)	Not mentioned	ESPB Group 0.25% ropivacaine 50 mg	After induction of anesthesia	6	4	30
								Control Group 0.9% saline 20 mL		11	5	32
Lin, H. 2022 (32)	–	12/30	Postherpetic neuralgia in elderly patients	Chronic pain/other	General anesthesia (sevoflurane + fentanyl balanced)	Bilateral (T5–T6)	Not mentioned	ESPB Group 0.375% ropivacaine 75 mg	After induction of anesthesia	5	–	42
		18/23						Control Group 0.9% saline 20 mL		13	–	41
Lin, Z. M. 2021 (31)	> 60 years	12/14	Postherpetic neuralgia in elderly patients	Chronic pain/other	General anesthesia (sevoflurane inhalation)	Unilateral (T8)	Not mentioned	ESPB group 0.4% ropivacaine 100 mg	Preoperative	2	0	26
		13/13						Control Group 0.9% saline 25 mL		5	1	26
Mohamed, R. M. 2023 (30)	20–65 years	10/20	Lumbar spine surgery	Spine	General anesthesia (sevoflurane 0.6 MAC + remifentanil infusion)	Bilateral (T4–T5)	Not mentioned	ESPB Group 0.375% ropivacaine 75 mg	After the induction of anesthesia	2	–	30
		12/18						Control Group 0.9% saline 20 mL		0	–	30
Mohasseb, A. M. 2024 (29)	20–60 years		Modified radical mastectomy	Breast	General anesthesia (TIVA)	Unilateral (T5)	Not mentioned	ESPB Group 0.5% bupivacaine 50 mg	After induction of anesthesia	9	–	36
								Control Group 0.9% saline 20 mL		11	–	36
Park, S. 2021 (27)	–		Mastectomy and immediate breast reconstruction with a tissue expander	Breast	General anesthesia (balanced GA with opioids)	Bilateral (T7–T8)	Not mentioned	ESPB Group 0.375% ropivacaine 112.5 mg	Before general anesthesia induction	6	4	29
								Control Group 0.9% saline 30 mL		11	3	29
Peng, J. 2023 (26)	60–79 years	18/14	Posterior lumbar spine surgery in elderly patients	Spine	General anesthesia (TIVA propofol + remifentanil)	Bilateral (L3–L4)	Pinprick test to detect analgesic block plane 30 min after block	ESPB Group 0.4% ropivacaine 80 mg	Before general anesthesia	3	–	32
		14/16						Control Group 0.9% saline 20 mL		6	–	30
Pişkin, Ö. 2022 (25)	18–75 years	12/28	Video-assisted thoracoscopic surgery	Thoracic/cardiac	General anesthesia (sevoflurane + remifentanil infusion)	Unilateral (T5)	Pinprick test to evaluate presence of sensory block before anesthesia induction	ESPB Group 0.25% bupivacaine 50 mg	Before the induction of general anesthesia	3	1	40
		24/12						Control Group 0.9% saline 20 mL		19	4	38
Sharipova, V. 2022 (24)	–	14/0	Kidney Transplant	Urologic	General anesthesia (balanced GA)	Unilateral (T8–T9)	Not mentioned	ESPB Group 0.125% bupivacaine 25 mg	Postoperative	2	–	14
		14/0						Control Group 0.9% saline 20 mL		8	–	14
Sifaki, F. 2023 (23)	35–85 years	3/5	Colorectal surgery	Abdominal/ abdominal wall	General anesthesia (sevoflurane balanced)	Bilateral (T4–T5)	Not mentioned	ESPB Group 0.2% ropivacaine 40 mg	Preoperative	2	–	8
		7/5						Control Group 0.9% saline 20 mL		2	–	12
Singh, S. 2020 (22)	18–65 years	17/3	Lumbar spine surgery	Spine	General anesthesia (sevoflurane + remifentanil)	Bilateral (T7)	Pin-prick sensation assessment in T6-L3 dermatomes every 5 min for 30 min; block failure if no sensation decrease	ESPB Group 0.5% bupivacaine 100 mg	Preoperative	0	–	20
		18/2						Control Group 0.9% saline 20 mL		2	–	20
Soni, S. 2024 (21)	18–60 years	0/32	Modified radical mastectomy	Breast	General anesthesia (TIVA propofol + remifentanil)	Bilateral (T7–T8)	Not mentioned	ESPB Group 0.2% ropivacaine 60 mg	Before general anesthesia	8	–	32
		0/32						Control Group 0.9% saline 30 mL		12	–	32
Tulgar, S. 2018 (20)	18–65 years	4/11	Laparoscopic cholecystectomy	Abdominal/ hepatobiliary	General anesthesia (balanced GA with opioids)	Bilateral (T7)	Not mentioned	ESPB Group 0.375% bupivacaine 75 mg	Before general anesthesia induction	1	–	15
		5/10						Control Group 0.9% saline 20 mL		4	–	15
van den Broek, R. J. C. 2021 (19)	–	9/11	Posterior lumbar interbody fusion surgery	Spine	General anesthesia (sevoflurane balanced)	Bilateral (L3–L4)	Not mentioned	ESPB Group 0.5% ropivacaine 200 mg	After induction of anesthesia	0	–	20
		7/13						Control Group 0.9% saline 40 mL		2	–	20
Wang, J. 2022 (18)	> 18 years	47/12	Open Liver Resection	Abdominal/ ab dominal wall	General anesthesia (TIVA propofol + remifentanil)	Unilateral (T8–T9)	Blunt 22-gauge needle test of anesthetized dermatomes at anterior midline, right midclavicular line, and right scapular line on POD1	ESPB Group 0.2% ropivacaine 60 mg	Preoperative	17	–	59
		47/12						Control Group 0.9% saline 30 mL		24	–	59
Wang, T. 2024 (17)	18–70 years	14/25	Video-assisted Thoracoscopic surgery	Thoracic/cardiac	General anesthesia (balanced GA for pediatric scoliosis)	Unilateral (T5)	Cold test with ice pack to assess anesthetized dermatomes at anterior and posterior axillary lines 24 h after surgery	ESPB group 0.5% ropivacaine 100 mg	Before induction of anesthesia	1	—-	39
		20/17						Control Group 0.9% saline 20 mL		3	–	37
Yao, Y. 2020 (55)	18–65 years	14/23	Video-assisted thoracic surgery	Thoracic/cardiac	General anesthesia (balanced GA)	Unilateral (T5)	Not mentioned	ESPB group 0.5% ropivacaine 125 mg	Upon arrival in the operating room	2	–	37
		15/23						Control Group 0.9% saline 25 mL		7	–	38
Yao, Y. 2019 (16)	18–65 years		Modified radical mastectomy	Breast	General anesthesia (TIVA propofol + remifentanil)	Unilateral (T4–T5)	Not mentioned	ESPB group 25 mL of 0.5% ropivacaine	Before induction of anesthesia	3	–	39
								Control Group 0.9% saline 25 mL		9	–	40
Yıldız Altun, A. 2020 (54)	–	7/16	Belt lipectomy	Abdominal/ abdominal wall	General anesthesia (sevoflurane balanced)	Bilateral (T7–T9)	Not mentioned	ESPB Group 0.25% bupivacaine 62.5 mg	Preoperative	6	2	23
		11/17						Control Group 0.9% saline 25 mL		16	9	28
Yu, Y. 2021 (15)	26–67 years	19/21	Posterior lumbar spinal surgery for lumbar fracture	Spine	General anesthesia (sevoflurane inhalation)	Unilateral (T5–T6)	Not mentioned	ESPB Group 0.25% bupivacaine 75 mg	After induction of anesthesia	4	3	40
		17/23						Control Group 0.9% saline 30 mL		17	16	40
Yuan, Z. 2022 (28)	1–3 years	12/18	Pediatric patients undergoing thoracoscopic lung lesion resection	Thoracic/cardiac	General anesthesia (balanced GA)	Bilateral (T7–T8)	Not mentioned	ESPB group 0.25% levobupivacaine 12.5 mg	After induction of anesthesia	4	2	30
		10/20						Control Group 0.9% saline 6 mL		11	9	30
Zhang, J. 2023 (14)	18–65 years	22/26	Video-assisted thoracoscopic lobectomy	Thoracic/cardiac	General anesthesia (desflurane + remifentanil)	Unilateral (T5)	Not mentioned	ESPB group 0.375% ropivacaine 112.5 mg	Before induction of anesthesia	4	–	48
		25/21						Control Group 0.9% saline 30 mL		13	–	46
Zhu, M. 2024 (13)	18–70 years	11/24	Arthroscopic shoulder surgery	Orthopedic	General anesthesia (TIVA propofol + remifentanil)	Unilateral (T8–T9)	Cold test for dermatomal sensory block evaluation (C4-T1 distribution) 20 min after block	ESPB group 0.25% ropivacaine 75 mg	Before induction of anesthesia	1	–	35
		15/20						Control Group 0.9% saline 30 mL		6	–	35
Zimmerer, A. 2022 (12)	> 18 years	24/10	Hip Arthroscopy	Orthopedic	Local anesthesia (awake outpatient ESPB only)	Unilateral (L3)	Not mentioned	ESPB group 0.375% ropivacaine 112.5 mg	Preoperative	2	–	34
		23/11						Control Group 0.9% saline 30 mL		3	–	34

### 2.4 Quality analysis of evidence

The GRADE (Grading of Recommendations, Assessment, Development, and Evaluation) approach is advised for determining the overall strength of evidence and for analyzing potential systematic bias as well as random errors. Studies were categorized as extremely low, low, moderate, or high-caliber, depending on bias risk, result inconsistency, lack of precision, publication bias, and the extent of treatment impacts.

### 2.5 Trial sequential analysis

Trial Sequential Analysis (TSA) was conducted utilizing the TSA software, version 0.9.5.10 beta, to ensure robust statistical evaluation and enhance the reliability of the results. The sufficiency of the patient count was evaluated, and TSA procedures were conducted to determine the need for additional research. The required information size was calculated, adjusting for diversity, which reflects variation among trials based on predicted sampling errors. TSA was employed to maintain the type I error rate at 5%, a threshold commonly accepted across numerous meta-analyses and systematic reviews. The essential data size was also ascertained (including an alpha error of 5% and a β error of 20%). Persuasive evidence is likely when the trial’s sequential monitoring threshold is exceeded before achieving the required level of information. If this threshold is not crossed, additional trials are usually needed to draw firm conclusions. In practical terms, TSA can be viewed as an analog of interim monitoring in a large clinical trial: it helps determine whether the currently available number of patients is already sufficient to support a reliable conclusion, or whether further studies are still required.

### 2.6 Outcome measures

A *Z*-test was employed to evaluate overall significance, with a *p*-value of less than 0.05 deemed to indicate statistical significance. A random effects model assessed the effectiveness of ESPB for nausea and vomiting by calculating the combined RD, accounting for factors such as administration timing, surgery type, local anesthetic type, and bupivacaine and ropivacaine dosages. For opioid consumption, the included trials reported different opioid agents (morphine, fentanyl, tramadol) and heterogeneous dosing regimens, routes of administration, rescue protocols and assessment windows. Converting all opioid doses to a single morphine milligram equivalent unit would require assumptions that were frequently not supported by study reporting and could introduce additional measurement error. Therefore, each opioid was analyzed separately and pooled using the SMD with 95% CI; different opioid agents were not combined in a single meta-analysis, and results should be interpreted accordingly. The combined SMD was also used to summarize VAS scores and the incidence of dizziness and pruritus, with VAS pooled at prespecified postoperative time windows (0–2, 4–6, 8–12, 24 h). Subgroup analyses were conducted according to local anesthetic type and dosage, concentration, surgical procedure, and timing of ESPB administration. We assessed potential publication bias and small-study effects using Begg’s rank correlation test and Egger’s regression test. In addition, we applied Duval and Tweedie’s trim-and-fill method based on the risk difference (RD) metric. The trim-and-fill analyses used the same model specifications as the primary meta-analyses (random-effects model for nausea and fixed-effect model for vomiting) to ensure consistency with the main forest plots. Sensitivity analyses focused on studies with low and uncertain risks to assess the robustness of the findings.

### 2.7 Meta-regression and leave-one-out sensitivity analysis

We performed leave-one-out sensitivity analyses by repeating the random-effects meta-analysis after omitting one trial at a time to evaluate the influence of individual studies on the pooled effect estimate and heterogeneity.

To further explore potential sources of between-study heterogeneity, we conducted random-effects meta-regression using study-level covariates extracted from the included trials (age, sex distribution, BMI, anesthetic technique (TIVA vs. non-TIVA), and baseline anti-emetic prophylaxis). Univariable and (when feasible) multivariable models were fitted with inverse-variance weighting under a random-effects framework. Missing moderator data were handled using available-case analysis.

## 3 Results

### 3.1 Study selection

**FIGURE 1:**
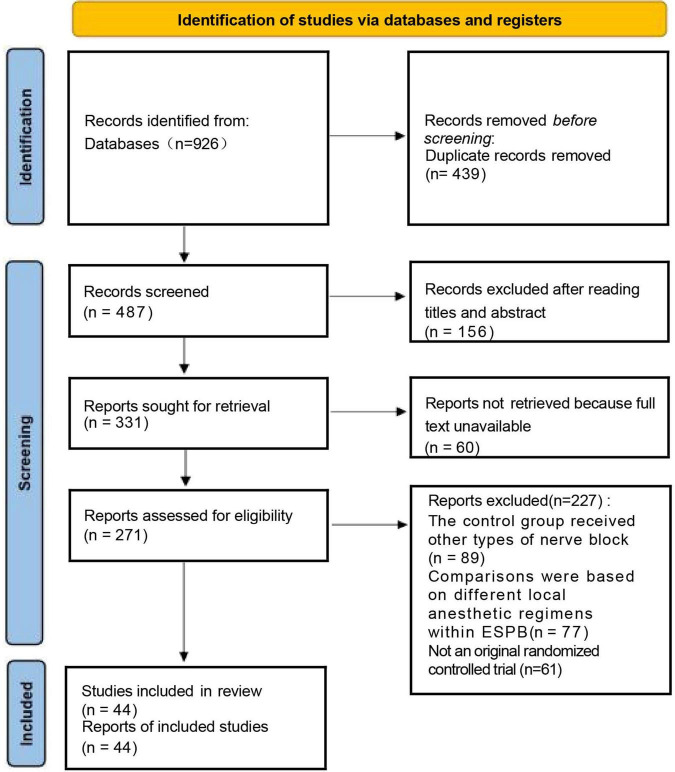
Flow diagram of the inclusion and exclusion process.

We identified 926 records through PubMed, Embase, and the Cochrane Library (Figure 1). After removal of 439 duplicates, 487 records remained for title and abstract screening, of which 156 were excluded as irrelevant. A further 60 records were excluded because the full text was unavailable. The remaining 271 full-text articles were assessed for eligibility, and 227 were excluded for the following reasons: the control group received other types of nerve blocks (*n* = 89), comparisons were based on different local anesthetic regimens within erector spinae plane block (*n* = 77), or the study was not an original randomized controlled trial (*n* = 61). Ultimately, 44 randomized controlled trials (12–55) were included in the final analysis.

### 3.2 Study characteristics

Of the literature reviewed, 44 randomized controlled trials (12–55) evaluated the effectiveness of ESPB in reducing the incidence of PONV (Table 1). Most studies were published between 2018 and 2024 and enrolled adult patients; three trials focused on pediatric or adolescent populations (lumbar fusion, scoliosis, and thoracoscopic lung lesion surgery), whereas the remaining trials included adults, often middle-aged or elderly. Spine procedures (12 trials) and abdominal or hepatobiliary/abdominal wall surgery (12 trials) were the most frequent surgical indications, followed by thoracic/cardiac surgery (7 trials), breast surgery (5 trials), urologic surgery (3 trials), orthopedic procedures (2 trials), chronic pain interventions for postherpetic neuralgia (2 trials), and one obstetric trial in cesarean delivery. Across studies, ESPB was almost uniformly performed under general anesthesia, with a few outpatient trials using ESPB as the main anesthetic technique. Most blocks were administered preoperatively or immediately after induction of anesthesia, predominantly at thoracic levels with smaller subsets at lumbar levels for lumbar spine and hip surgery. Bupivacaine and ropivacaine were the most commonly used local anesthetics, with concentrations typically ranging from 0.2 to 0.5% and volumes around 20–30 mL.

### 3.3 The methodological quality of the included studies

**FIGURE 2:**
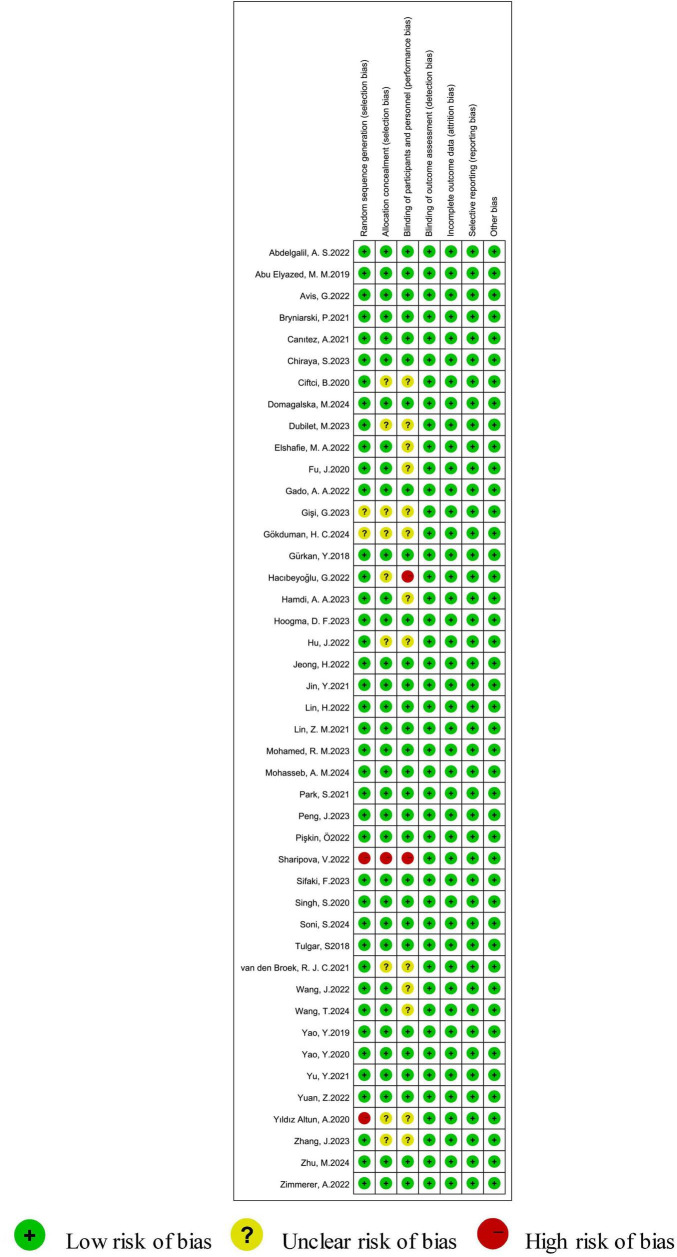
Summary of the risk of bias of the included studies.

Forty-four trials showed a minimal general risk of bias. Among these, six studies utilized computer-generated random numbers, 22 applied random number tables, and sixty used sealed envelopes as their method for randomization. Two studies failed to specify blinding methods, prompting a “high risk of bias” assessment. We classified the risk of bias as “unclear” in cases where certain studies did not explicitly provide details on allocation concealment. All studies reported conclusions without dropouts and included all endpoints specified in the Methods section, suggesting potential reporting bias. Additional biases may exist across all studies. A summary of possible bias sources is presented in Figure 2.

### 3.4 Quality of evidence

The GRADE ratings for nausea and vomiting are summarized in Table 2. For nausea, we started from high-certainty evidence because all contributing studies were randomized trials and downgraded by one level for risk of bias (two trials lacked blinding and one trial had unclear allocation concealment) and by one level for inconsistency (I^2^ values in the moderate-to-high range), resulting in an overall rating of moderate certainty. We did not downgrade for indirectness or publication bias, and imprecision was judged as not serious because the confidence interval around the pooled effect did not cross the line of no effect. For vomiting, the overall certainty was rated as high: the risk of bias was generally low, heterogeneity was low to moderate, and the confidence interval was relatively narrow and compatible with a clinically important reduction in vomiting. Although the total number of participants was below 400 in some comparisons, the trial sequential analysis suggested that the optimal information size had been reached. In both outcomes, very strong or strong associations (risk ratios well below 1.0) were interpreted as factors that may increase, rather than decrease, confidence in the effect estimates; therefore, “RR < 0.2” is no longer listed as a reason for downgrading.

**TABLE 2:** GRADE summary of efficacy of ESPB on PONV.

Quality assessment							No of patients		Effect		Quality	Importance
No of studies	Design	Risk of bias	Inconsistency	Indirectness	Imprecision	Other considerations	Continuous	Control	Relative (95% CI)	Absolute		
**Fentanyl (μ g)**												
4	Randomized trials	Serious^1^	Serious^2^	No serious indirectness	Serious^3^	Very strong association^4^	97	102	–	SMD 2.96 lower (5.13–0.79 lower)	ÅÅÅO Moderate	Important
**Morphine 1–48 h(mg)**												
5	Randomized trials	No serious risk of bias	Serious	No serious indirectness	Serious^3^	None	124	124	–	SMD 0.86 lower (1.54–0.18 lower)	ÅÅOO Low	Important
**Tramadol(mg)**												
4	Randomized trials	No serious risk of bias	Serious^2^	No serious indirectness	Serious^3^	Reporting bias^5^ very strong association^4^	95	97	–	SMD 1.43 lower (2.32–0.55 lower)	ÅÅÅO Moderate	Important
**Dizziness**												
5	Randomized trials	Serious	No serious inconsistency	No serious indirectness	Serious^3^	Strong association^6^	6/169 (3.6%)	15/168 (8.9%)	RR 0.43 (0.18–1.02)	51 fewer per 1000 (from 73 fewer to 2 more)	ÅÅÅO Moderate	Critical
								9.8%		56 fewer per 1000 (from 80 fewer to 2 more)		
**Itching**												
7	Randomized trials	No serious risk of bias	No serious inconsistency	No serious indirectness	Serious^3^	Reporting bias strong association^6^	22/255 (8.6%)	56/254 (22%)	RR 0.39 (0.25–0.61)	134 fewer per 1000 (from 86 fewer to 165 fewer)	ÅÅÅO Moderate	Important
								20%		122 fewer per 1000 (from 78 fewer to 150 fewer)		
**Nausea**												
43	Randomized trials	Serious^1^	Serious^2^	No serious indirectness	No serious imprecision	Reporting bias^5^ very strong association^4^	199/1362 (14.6%)	434/1352 (32.1%)	See comment	164 fewer per 1000 (from 119 fewer to 209 fewer)	ÅÅÅO Moderate	Critical
								30.6%		156 fewer per 1000 (from 113 fewer to 199 fewer)		
								22.5%		101 fewer per 1000 (from 58 fewer to 142 fewer)		
**Quality assessment**							**No of patients**		**Effect**		**Quality**	**Importance**
**No of studies**	**Design**	**Risk of bias**	**Inconsistency**	**Indirectness**	**Imprecision**	**Other considerations**	**Continuous**	**Control**	**Relative** **(95% CI)**	**Absolute**		
**Vomiting**												
12	Randomized trials	Serious^1^	No serious inconsistency	No serious indirectness	Serious^3^	Very strong association^4^	32/394 (8.1%)	80/397 (20.2%)	See comment	119 fewer per 1000 (from 71 fewer to 169 fewer)	ÅÅÅÅ HIGH	CRITICAL
								17.8%		105 fewer per 1000 (from 62 fewer to 150 fewer)		

^1^Two studies lacked blinding and one study had unclear allocation concealment (reason for downgrading risk of bias).

^2^I^2^ values indicated moderate or high thresholds for statistical heterogeneity (reason for downgrading inconsistency).

^3^Total population size is less than 400 in some comparisons (reason for downgrading imprecision when applicable).

^4^Very strong association (RR < 0.2), which according to GRADE may increase, rather than decrease, the certainty of evidence when other domains are not seriously compromised.

^5^Presence of manufacturer-sponsored studies, suggesting a potential risk of reporting or publication bias.

^6^Strong association (RR < 0.5), which may support increased confidence in the effect estimate when consistent with other domains.

### 3.5 Results of meta-analysis

#### 3.5.1 ESPB on PONV

**FIGURE 3:**
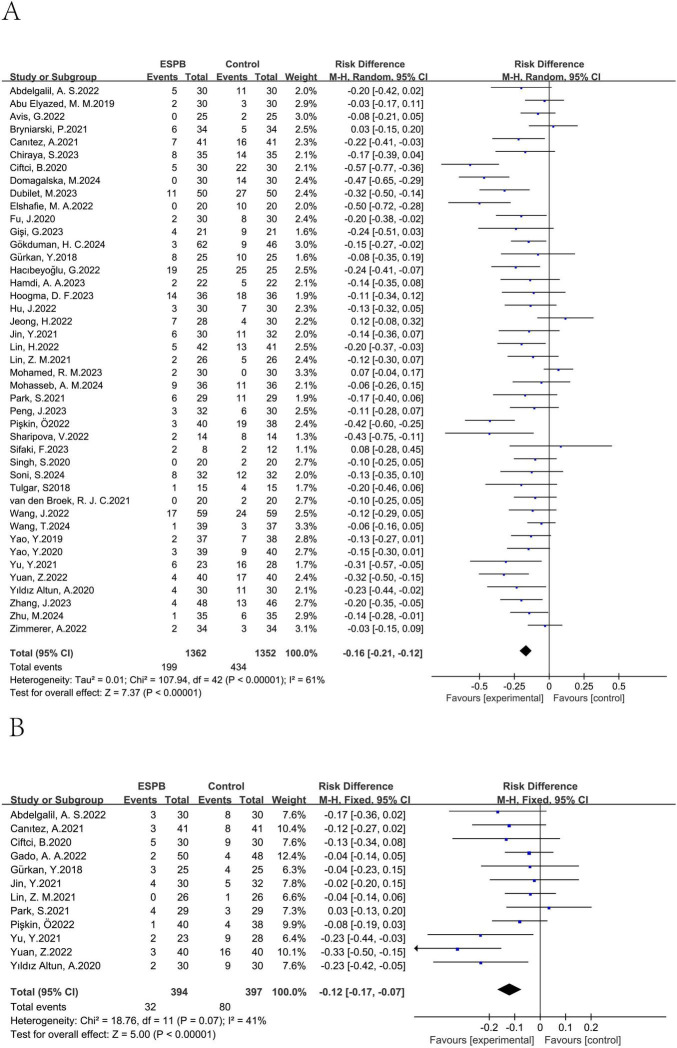
Results of the incidence of nausea **(A)** and vomiting **(B)**.

**FIGURE 4:**
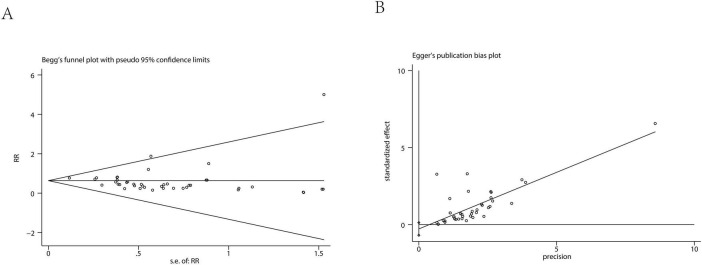
Results of the Begg’s **(A)** test and Egger’s **(B)** test of nausea.

**FIGURE 5:**
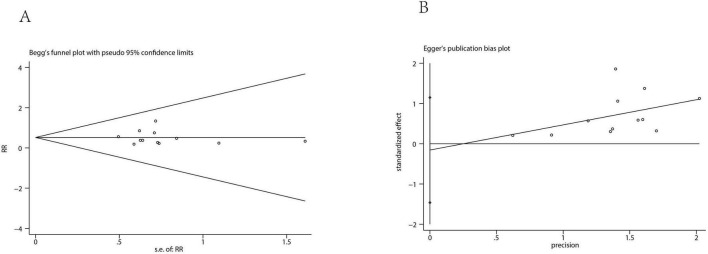
Results of the Begg’s test **(A)** and Egger’s **(B)** test of vomiting.

A total of 43 studies (12–41, 43–55) involving 2,714 participants evaluated the effectiveness of preventing nausea. Additionally, 12 studies (15, 25, 27, 28, 31, 33, 39, 42, 47, 49, 53, 54) with 791 patients assessed vomiting about ESPB versus no ESPB. The incidence of nausea in the ESPB group was significantly lower than in the control group (combined RD = -0.16, 95% CI: -0.21 to -0.12), and the rate of vomiting also decreased compared to the control group (combined RD = -0.12, 95% CI: -0.17 to -0.07) (Figure 3). Results from Begg’s test (*P* = 0.884) and Egger’s test (*P* = 0.180) suggested that there was no significant publication bias for nausea, and similarly, Begg’s test (*P* = 0.784) and Egger’s test (*P* = 0.156) indicated no significant publication bias for vomiting in the ESPB group. This suggests a reliable evaluation of nausea outcomes. For both nausea and vomiting, Begg’s and Egger’s tests indicated no significant publication bias (nausea: Begg’s *P* = 0.884, Egger’s *P* = 0.180; vomiting: Begg’s *P* = 0.784, Egger’s *P* = 0.156) (Figures 4, 5), supporting a reliable evaluation of outcomes in the ESPB group.

Trim-and-fill analysis further confirmed the robustness of the results. For nausea, a random-effects RD model imputed three potentially missing studies, but the adjusted pooled effect size remained similar to the original estimate, with no change in the direction or statistical significance. For vomiting, a fixed-effect RD model imputed zero studies, indicating no missing studies were needed to restore funnel plot symmetry and that the pooled effect estimate was unchanged after adjustment (Supplementary Figures 3, 4).

##### 3.5.1.1 Meta-regression and leave-one-out sensitivity analyses

For vomiting, leave-one-out analyses similarly showed robust results (pooled RD range: -0.113 to-0.072; Supplementary Tables 3, 5; Supplementary Figure 2). Leave-one-out analyses demonstrated that the pooled effect remained stable after omitting any single trial (pooled RD range: -0.170 to -0.153), with no change in the direction of effect (Supplementary Table 4; Supplementary Figure 1).

For nausea, meta-regression did not identify statistically significant effect modification by age, sex distribution, BMI, anesthetic technique, or baseline anti-emetic prophylaxis (all *P* > 0.05; Supplementary Tables 1, 2).

Furthermore, factors influencing nausea and vomiting were examined through subgroup analysis:

#### 3.5.2 Operation type

**FIGURE 6:**
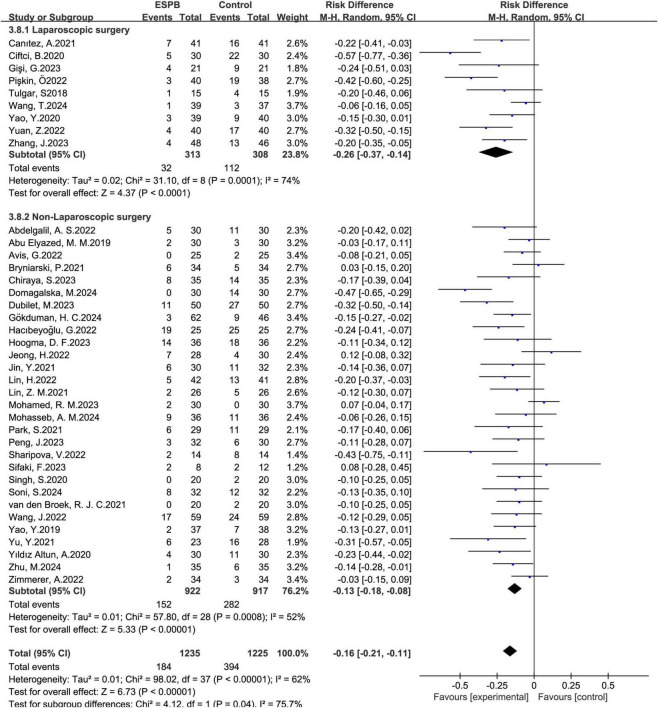
Results of the subgroup of operation type.

ESPB markedly reduced the nausea rates in laparoscopic surgery; the pooled RD of 9 (14, 17, 20, 25, 28, 41, 47, 49, 55) trials was -0.26, with a 95% confidence interval (CI) of -0.37 to -0.14. Moreover, in non-laparoscopic surgery, the pooled RD of 29 trials (12, 13, 15, 16, 18, 19, 21–24, 26, 27, 29–34, 36, 38, 40, 45, 46, 48, 50–54) was -0.13, with a 95% CI of -0.18 to -0.08 (Figure 6).

#### 3.5.3 Type of local anesthetic

**FIGURE 7:**
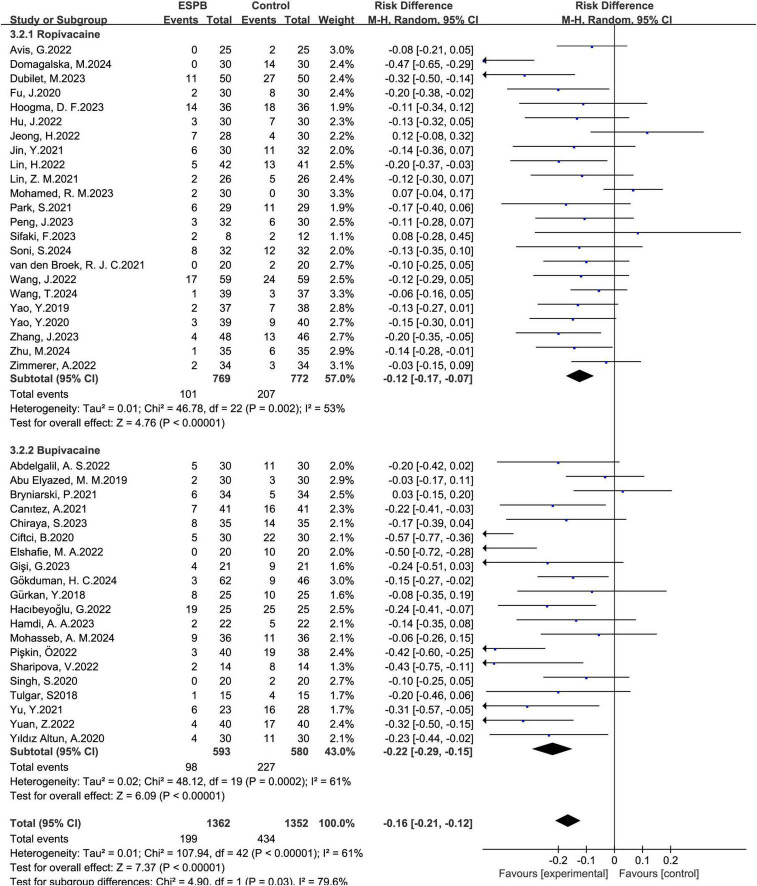
Results of the subgroup of local anesthetic type.

ESPB markedly decreased nausea occurrences with ropivacaine as the local anesthetic (pooled RD of 23 (12–14, 16–19, 21, 23, 26, 27, 30–36, 43, 45, 46, 51, 55) trials: -0.12, 95% CI: -0.17 to -0.07), and bupivacaine (pooled RD of 20 (15, 20, 22, 24, 25, 28, 29, 37, 40, 41, 44, 47–50, 52–54) trials: -0.22, 95% CI: -0.29 to -0.15) (Figure 7).

#### 3.5.4 Local anesthetic dosage

**FIGURE 8:**
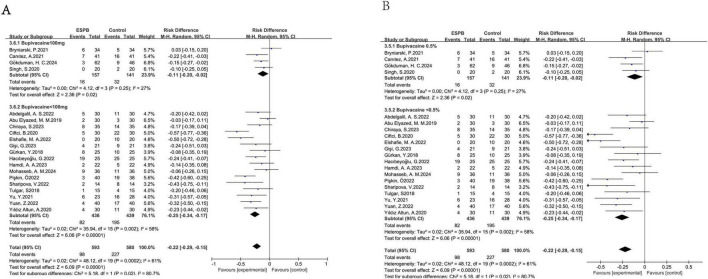
Results of the local anesthetic of bupivacaine dosage **(A)** and concentration **(B)**.

**FIGURE 9:**
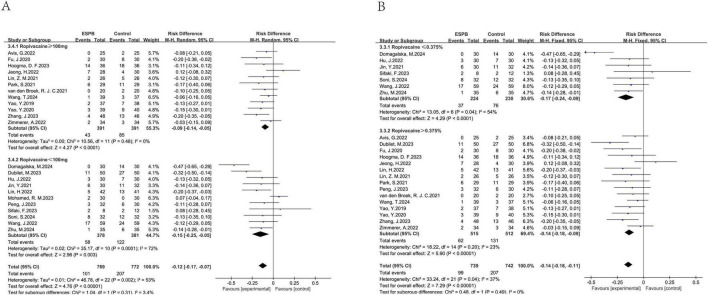
Results of the local anesthetic of ropivacaine dosage **(A)** and concentration **(B)**.

ESPB significantly reduced the occurrences of nausea. When the dosage of ropivacaine was 100 mg or more (the pooled risk difference of twelve trials (12, 14, 16, 17, 19, 27, 31, 34, 36, 43, 51, 55) was -0.09, with a 95% confidence interval of -0.14 to -0.05) and the dosage of bupivacaine was 100 mg (the pooled risk difference of four trials (22, 40, 49, 50) was -0.11, with a 95% confidence interval of -0.20 to -0.02), as well as when the ropivacaine dosage was less than 100 mg (the pooled risk difference of eleven trials (13, 18, 21, 23, 26, 30, 32, 33, 35, 45, 46) was -0.15, with a 95% confidence interval of -0.25 to -0.05) and the bupivacaine dosage was less than 100 mg (the pooled risk difference of sixteen (15, 20, 24, 25, 28, 29, 37–39, 41, 44, 47, 48, 52–54) trials was -0.25, with a 95% confidence interval of -0.34 to -0.17), this effect was observed (Figures 8A, 9A).

#### 3.5.5 Local anesthetic concentration

ESPB significantly reduced the occurrences of nausea. When the ropivacaine level was ≤ 0.375% (the pooled risk difference of seven trials (13, 18, 21, 23, 33, 35, 46) was -0.17, with a 95% confidence interval of -0.24 to -0.09) and the bupivacaine concentration was 0.5% (the pooled risk difference of four trials (22, 40, 49, 50) was -0.11, with a 95% confidence interval of -0.20 to -0.02), as well as when the ropivacaine concentration was > 0.375% (the pooled risk difference of fifty (12, 14, 16, 17, 19, 26, 27, 31, 32, 34, 36, 43, 45, 51, 55) trials was -0.14, with a 95% confidence interval of -0.18 to -0.09) and the bupivacaine concentration was < 0.5% (the pooled risk difference of sixteen (15, 20, 24, 25, 28, 29, 37–39, 41, 44, 47, 48, 52–54) trials was -0.25, with a 95% confidence interval of -0.34 to -0.17), this effect was observed (Figures 8B, 9B).

#### 3.5.6 Time of administration

**FIGURE 10:**
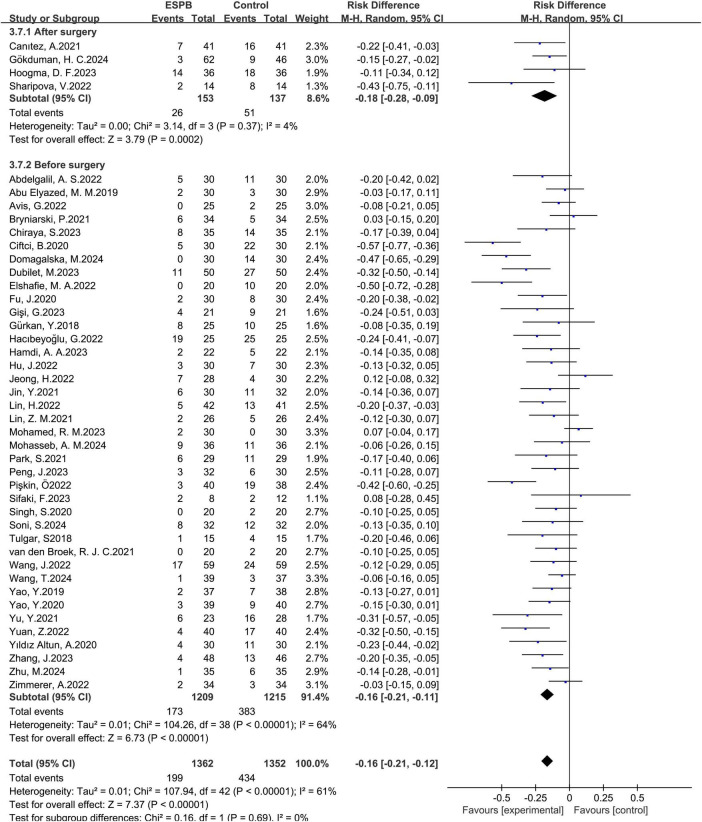
Results of the subgroup of time of administration.

ESPB significantly reduced the occurrences of nausea. When the timing of administration was before surgery, the pooled risk difference of 39 trials (12–20, 22, 23, 26–35, 37–39, 41, 43, 44, 46–48, 50–53, 55) was -0.16, with a 95% confidence interval of -0.21 to -0.11. When the administration was after surgery, the pooled risk difference of 4 trials (24, 36, 40, 49) was -0.18, with a 95% confidence interval of -0.28 to -0.09 (Figure 10).

#### 3.5.7 VAS movement at 0–2, 4–6, 8–12, and 24 h

**FIGURE 11:**
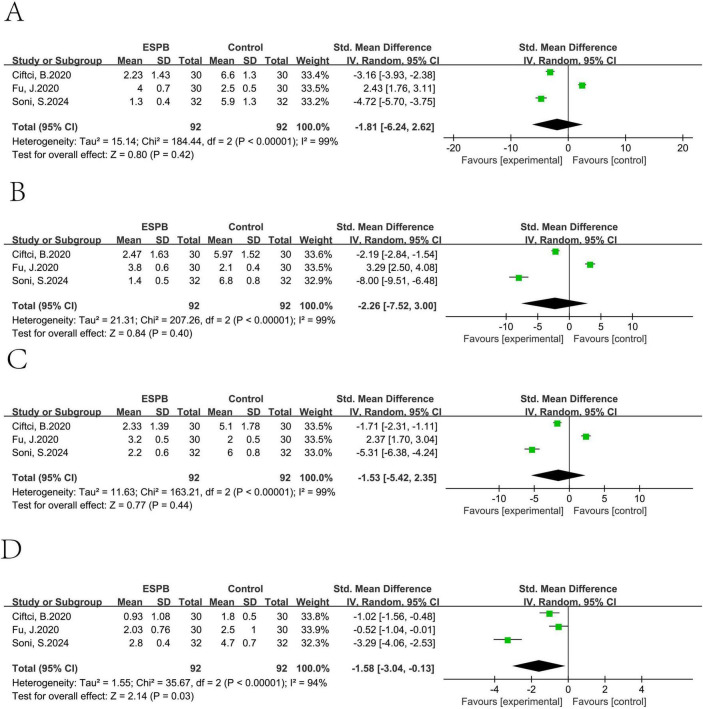
Results of VAS movement at 0–2h **(A)**, 4–6h **(B)**, 8–12h **(C)** and 24h **(D)**.

ESPB could reduce the VAS movement at 24 h (pooled SMD of three trials (21, 43, 47): -1.58, 95% CI: -3.04 to -0.13), but not at 0–2 h (pooled SMD of three trials (21, 43, 47): -1.81, 95% CI: -6.24 to 2.62), at 4–6 h (pooled SMD of three trials (21, 43, 47): -2.26, 95% CI: -7.52 to 3.00), and 8–12 h (pooled SMD of three trials (21, 43, 47): -1.53, 95% CI: -5.42 to 2.35) (Figure 11).

#### 3.5.8 Consumption of morphine, fentanyl, and tramadol

**FIGURE 12:**
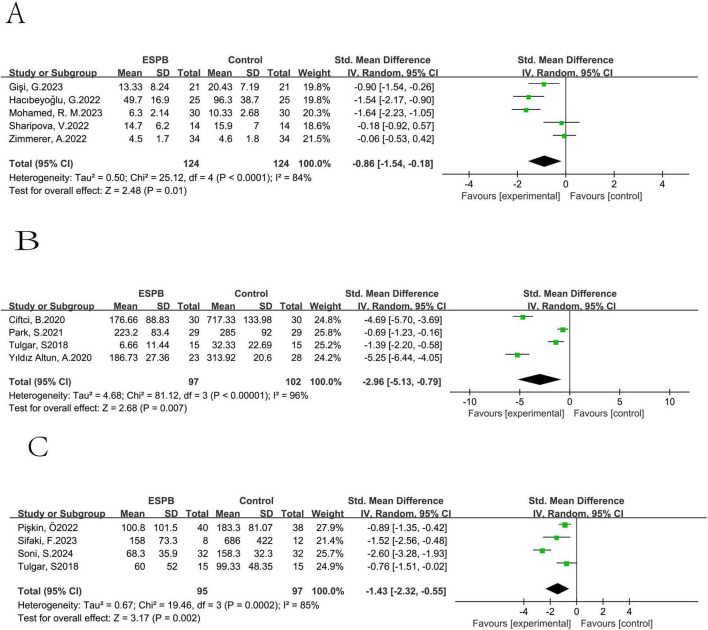
Results of consumption of morphine **(A)**, fentanyl **(B)** and tramadol **(C)**.

ESPB reduced the consumption of morphine [pooled SMD of five trials (12, 24, 30, 38, 41): -0.86, 95% CI: -1.54 to -0.18; Figure 12A], fentanyl [pooled SMD of four trials (20, 27, 47, 54): -2.96, 95% CI: -5.13 to -0.79; Figure 12B] and tramadol [pooled SMD of four trials (20, 21, 23, 25): -1.43, 95% CI: -2.32 to -0.55; Figure 12C].

#### 3.5.9 Probability of dizziness, itching

**FIGURE 13:**
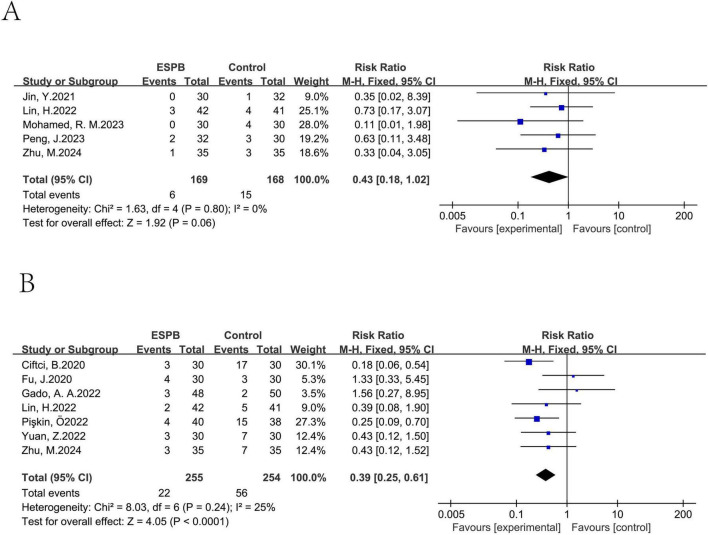
Results of probability of dizziness **(A)**, itching **(B)**.

ESPB was found to reduce the occurrence of dizziness (pooled risk ratio (RR) of five trials (13, 26, 30, 32, 33): 0.43, 95% CI: 0.18–1.02). It also reduced the probability of itching [pooled RR of seven trials (13, 25, 28, 32, 42, 43, 47): 0.39, 95% CI: 0.25–0.61; Figure 13].

### 3.6 Trial sequential analysis

**FIGURE 14:**
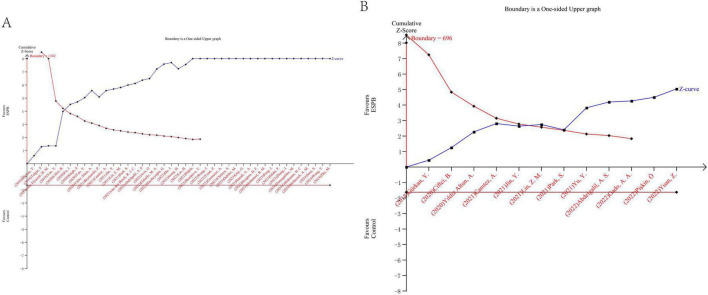
Trial sequential analysis results. **(A)** Trial sequential analysis for nausea. **(B)** Trial sequential analysis for vomiting.

Our trial sequential analysis (TSA) provides a thorough and focused evaluation of nausea and vomiting. For nausea, data from 43 randomized controlled trials involving 2,714 patients were included; for vomiting, 12 trials with 791 patients were analyzed. The required information size was adjusted for between-study diversity with a type I error of 5% and a type II error of 20%. For both nausea and vomiting, the cumulative Z curve crossed the trial sequential monitoring boundary and reached the diversity-adjusted required information size (Figure 14). In simpler clinical terms, this means that the current number of patients is large enough to conclude, with reasonable confidence, that ESPB reduces the incidence of nausea and vomiting, and that the risk of these findings being false positives due to random error or repeated significance testing is low. Conversely, TSA did not suggest that a substantial number of additional trials would be needed to confirm a benefit of similar magnitude.

## 4 Discussion

PONV is a common complication after anesthesia and represents a major challenge in the perioperative setting. It not only impairs patient comfort but can also delay recovery and lead to serious complications such as airway obstruction, dehydration, electrolyte disturbances, wound complications, increased pain and prolonged hospital stay (56, 57). PONV has been reported in up to 80% of high-risk patients and in approximately 30% of all surgical patients (58), thereby increasing healthcare utilization and costs (59). Although pharmacological prophylaxis with agents such as ondansetron and dexamethasone is widely used and often effective, a substantial proportion of patients still experience nausea and vomiting despite antiemetic therapy (60, 61). Consequently, there is growing interest in non-pharmacological strategies to prevent PONV. In this context, our review evaluates the role of the erector spinae plane block (ESPB) as a regional anesthetic technique that may reduce PONV while providing effective postoperative analgesia. The key findings of this meta-analysis can be summarized as follows. First, patients who received ESPB had a significantly lower incidence of PONV, suggesting that ESPB may effectively mitigate this common complication. Second, ESPB reduced pain scores on movement at 24 h. Third, ESPB reduced perioperative consumption of morphine, fentanyl and tramadol. Fourth, ESPB was associated with a lower incidence of dizziness and pruritus.

In the past few decades, opioid drugs have largely mitigated acute surgical pain. Despite their high efficacy in managing pain during surgery, opioids can be linked to conditions like dizziness, urinary retention, ileus, PONV, delirium, sedation, constipation, tolerance, and respiratory depression (62, 63). Consequently, clinicians are striving to reduce perioperative opioid consumption. The development of multimodal analgesic approaches based on regional anesthesia has been swift in recent times. The adoption of these strategies may lower opioid use during surgeries, alleviate postoperative pain, and improve recovery outcomes. Recently, ESPB has become increasingly popular in perioperative care, demonstrating efficacy in comprehensive pain management throughout surgery (64). The use of ultrasound-assisted ESPB has the potential to enhance both precision and safety (65). Post-surgical recovery advocates for multimodal pain relief during the perioperative period lessens opioid consumption and accelerates the recuperation of patients undergoing surgery (66). As a relatively new regional block, the mechanism of ESPB remains to be studied (67).

ESPB is a regional anesthetic technique in which local anesthetic is injected into the fascial plane deep to the erector spinae muscle and superficial to the transverse processes (68). Anatomical and imaging studies suggest that the injectate can spread to the paravertebral space and along the dorsal and ventral rami, providing multi-dermatomal analgesia. By improving both somatic and visceral pain control and reducing opioid requirements, ESPB may help prevent opioid-related adverse effects, promote bowel function recovery, and diminish PONV, thereby facilitating faster postoperative recovery (69). When comparing study outcomes, it is essential to consider factors like anesthetic type, dosage, concentration, duration, and surgical techniques.

The commencement of the sensory block seemed to be gradual, potentially taking as long as 60 min to achieve its peak impact; thus ideally, the block should be administered earlier, allowing sufficient time for pain relief to begin. Administering ESPB before surgery resulted in enhanced pain alleviation and a decrease in the opioid dosage during the perioperative period. Our results indicate that most ESPB is performed preoperatively, allowing sufficient time for analgesic effects to take place and for reducing opioid usage and associated side effects (70). Due to the scarcity of studies on ESPB administered postoperatively, our subgroup analysis regarding the effects of ESPB on PONV did not find that preoperative blocks have a distinct advantage. Further research is needed to confirm its effects.

The efficacy of the ESPB in minimizing PONV is strongly influenced by the specific surgical procedure involved. In procedures involving the abdominal cavity, such as laparoscopic surgeries, one way that ESPB may reduce PONV is by inhibiting sensory nerves to the abdominal organs, which helps to relieve visceral pain (71). Conversely, in orthopedic surgeries, such as hip or knee replacements, the mechanism may involve alleviating somatic pain, which can indirectly reduce the stress response and subsequent nausea (12). The ESPB influences PONV differently in laparoscopic surgeries, where abdominal insufflation can heighten PONV. ESPB helps by blocking sensory innervation to the abdominal wall and viscera, reducing visceral pain and stress (72). In contrast, open surgeries, which are non-laparoscopic, tend to cause greater tissue damage and are associated with distinct pain profiles compared to minimally invasive procedures. While ESPB provides analgesic benefits, its impact on PONV is often less pronounced due to higher pain and stress levels, along with increased opioid usage that contributes to nausea. We did not observe a significant correlation between nausea and vomiting and the type of surgery, which may be attributed to the limited number of studies available. Higher concentrations and dosages enhance analgesia by more effectively blocking sensory nerve transmission, reducing visceral pain and the stress response associated with PONV. In contrast, lower concentrations may lead to inadequate pain relief, increasing the risk of PONV. Thus, optimizing the concentration and dosage of local anesthetics in ESPB is essential for maximizing their efficacy in preventing PONV (73). We did not observe any differences in the concentration or dose subgroups, which may be due to the limited number of studies included in the literature.

The ESPB has the potential to alleviate itching and dizziness by delivering effective analgesia, which helps to relieve pain and discomfort during medical procedures (74). It blocks sensory nerve transmission in the thoracic region, potentially decreasing inflammatory mediators that trigger itching, while also reducing stress and anxiety related to surgery, which helps alleviate dizziness (16). Overall, ESPB enhances comfort and decreases the incidence of these symptoms in surgical patients. We also found that ESPB can reduce the occurrence of dizziness and itching. Our findings indicate that ESPB is effective in reducing pain, lowering VAS scores, and minimizing opioid consumption post-surgery. Our meta-analysis revealed that the use of ESPB decreased the requirement for opioids during both the intraoperative and postoperative periods, subsequently reducing the incidence of PONV. These findings align with the results of a previous study (12).

An important finding of our meta-analysis is that several pooled estimates were accompanied by moderate to high statistical heterogeneity. This variability is not unexpected given the wide range of clinical settings in which ESPB has been used, but it limits the strength of any single summary effect and warrants careful interpretation. We therefore attempted to explore potential sources of heterogeneity through a series of subgroup analyses. Specifically, we stratified studies by surgical category (laparoscopic vs. non-laparoscopic procedures), type of local anesthetic (ropivacaine vs. bupivacaine), local anesthetic dose (≥ 100 mg vs. < 100 mg), concentration (e.g., ropivacaine ≤ 0.375% vs. > 0.375%, bupivacaine 0.5% vs. < 0.5%), and timing of ESPB administration (pre- vs. postoperative). Across these subgroups, ESPB consistently demonstrated a reduction in PONV, opioid consumption, and pain scores, suggesting that the beneficial effect is robust across a variety of clinical scenarios. However, the I^2^ values frequently remained in the moderate range, indicating that these variables only partially explain the observed heterogeneity and that additional, unmeasured factors are likely to be involved.

Beyond the factors we formally analyzed, several clinically important variables likely contributed to residual heterogeneity but were unsuitable for quantitative synthesis. Anesthetic protocols differed (balanced volatile vs. total intravenous techniques, variable opioid-sparing strategies), as did baseline antiemetic regimens, which ranged from standardized multimodal prophylaxis to poorly described or selective use. ESPB techniques also varied (thoracic vs. lumbar, single- vs. two-level, unilateral vs. bilateral), and PONV outcomes were defined and assessed at different time points. Because reporting was limited and subgroup sizes were small, further subgroup analyses or meta-regression were not feasible, underscoring the need for better-standardized future trials.

Although funnel plot–based methods, including Begg’s test, Egger’s test, and trim-and-fill analysis, are commonly used to explore publication bias, their statistical power may be limited when the number of included studies is relatively small. Therefore, the absence of imputed studies, particularly for the vomiting outcome, should not be interpreted as definitive evidence of no publication bias. To further reduce the risk of false-positive findings arising from sparse data and repeated significance testing, we conducted TSA. TSA provides more conservative monitoring boundaries and required information sizes, thereby mitigating random errors and strengthening the robustness of statistically significant results in cumulative meta-analyses.

The mechanisms by which ESPB may reduce PONV are likely multifactorial and extend beyond simple somatic analgesia. Anatomical and imaging studies have shown that injectate can spread from the erector spinae plane into the paravertebral and, in some cases, epidural space, with blockade of dorsal and ventral rami, rami communicants and the sympathetic chain (75, 76). This pattern of spread supports both visceral and somatic analgesia, attenuating nociceptive input from thoracic and upper abdominal organs to the spinal cord and, ultimately, to brainstem emetic centers. By providing more effective visceral pain control, ESPB may diminish vagal and spinal afferent activation of the nucleus tractus solitarius and area postrema, thereby lowering the propensity for nausea and vomiting (49). In parallel, the opioid-sparing effect of ESPB reduces exposure to a well-established pharmacological trigger of PONV, and the mitigation of the surgical stress response may help preserve gastrointestinal motility and perfusion (77). Taken together, these mechanistic data provide a biologically plausible explanation for the consistent association between ESPB, reduced opioid consumption and the lower incidence of PONV observed in our meta-analysis.

Clinical implications and integration into ERAS: Enhanced Recovery After Surgery (ERAS) programs emphasize multimodal, opioid-minimizing analgesia and routine PONV prophylaxis to facilitate early ambulation and resumption of oral intake. Against this background, our results support considering ESPB as an adjunct regional technique within ERAS pathways, especially for laparoscopic and abdominal/hepatobiliary surgery, where it may lessen opioid exposure, improve functional pain control during movement, and reduce nausea/vomiting. In practice, ESPB can be delivered under ultrasound guidance before surgery or soon after induction, in combination with scheduled non-opioid analgesics and standard antiemetic regimens. Future ERAS-focused trials should standardize accompanying interventions and evaluate recovery-centered outcomes (e.g., time to first oral intake, mobilization milestones, and discharge readiness) to clarify the added benefit of ESPB within ERAS care.

### 4.1 Limitations and suggestions for practice

This meta-analysis has several limitations that should be acknowledged when interpreting the findings. First, although we included a relatively large number of randomized trials, the statistical heterogeneity for several outcomes was moderate to high, and could not be fully explained by our predefined subgroup analyses based on surgical type, local anesthetic agent, dose, concentration, and timing of ESPB. Important clinical variables—including anesthetic protocols (e.g., volatile versus total intravenous anesthesia), baseline antiemetic regimens, ESPB level (thoracic vs. lumbar), and unilateral versus bilateral blocks—were often incompletely reported or highly variable across studies, which precluded more detailed subgroup analyses or robust meta-regression and likely contributed to residual heterogeneity. Second, across included trials, ESPB technique reporting was inconsistent, with most studies not reporting sensory block verification, dermatomal spread, or ultrasound confirmation of injectate distribution. As blocks were frequently performed after induction of general anesthesia, formal dermatomal testing was often not feasible, and unrecognized block failure cannot be excluded. This lack of standardized technique reporting may have contributed to clinical heterogeneity and limited reproducibility and clinical translation. To address this limitation, future trials should predefine block success criteria and systematically report verification methods, such as dermatomal sensory mapping when feasible, ultrasound-confirmed fascial spread or injectate distribution, or validated alternative approaches. Adoption of standardized reporting frameworks, including ESPB-specific checklists or established perioperative reporting guidelines such as RELIEF, would improve methodological transparency and comparability across studies. Third, Opioid consumption was reported using different agents, routes, and assessment windows, and we synthesized morphine, fentanyl and tramadol separately without converting doses to a common morphine milligram equivalent unit. While this approach avoids potentially unreliable assumptions required for equianalgesic conversion, it limits interpretability and precludes a single pooled estimate across opioids. Future trials should report cumulative opioid use in morphine milligram equivalents with clearly defined routes and time windows to facilitate comparability across studies. Moreover, definitions and reporting formats for PONV were not fully consistent: some trials reported composite PONV, whereas others reported nausea and vomiting as separate outcomes, and postoperative assessment times varied across studies (Supplementary Table 6). Although we attempted to harmonize these differences by analyzing composite outcomes as reported and choosing the time interval closest to 24 h, these methodological variations may still have attenuated or exaggerated the true effect of ESPB on PONV. Finally, although the overall sample size was sufficient according to trial sequential analysis for the primary outcomes, several secondary outcomes were informed by only a small number of trials. In particular, cumulative opioid consumption and VAS scores at each postoperative time window were based on limited data, with heterogeneous opioid regimens and non-uniform VAS assessment times, which reduces precision and limits the generalizability of these secondary findings. Future large, high-quality randomized trials with standardized reporting of anesthetic and antiemetic protocols, detailed ESPB characteristics (level, laterality, dose and concentration), harmonized PONV assessment (e.g., using ISO-PUF or I-FEED tools), and more uniform definitions of pain and opioid outcomes are needed to clarify the sources of heterogeneity and further refine the clinical indications for ESPB in PONV prevention.

## 5 Conclusion and recommendations

In conclusion, ESPB appears to provide effective postoperative analgesia while reducing the need for opioid analgesics, thereby lowering the risk of PONV and other opioid-related adverse effects. Trial sequential analysis indicated that the cumulative evidence has reached the required information size and crossed the monitoring boundary, suggesting that additional trials with similar designs are unlikely to overturn the conclusion that ESPB reduces postoperative nausea and vomiting. Overall, ESPB may be regarded as a safe regional block with a relatively low incidence of complications and represents a valuable option in multimodal perioperative care.

## Data Availability

The original contributions presented in the study are included in the article/Supplementary material, further inquiries can be directed to the corresponding author.
