# Supplementary figures and images for: Erector spinae plane block reduces postoperative nausea and vomiting: a systematic review and meta-analysis of 44 randomized trials
Source: Front Med (Lausanne). 2026 Jan 16;12:1749998. doi: 10.3389/fmed.2025.1749998 (PMC12855405; doi:10.3389/fmed.2025.1749998)

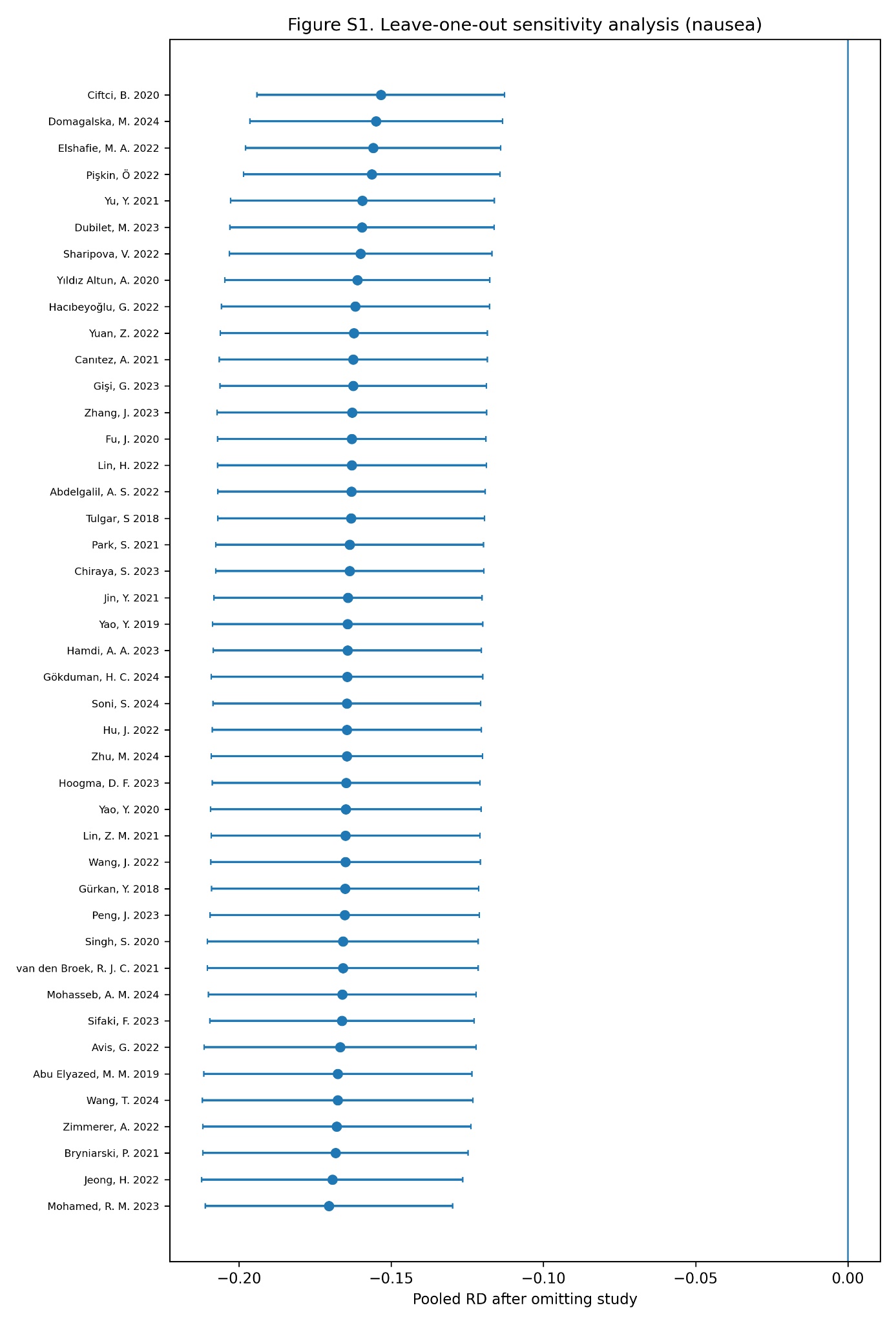

Supplement: Supplementary file 1 [file Image_1.jpg]

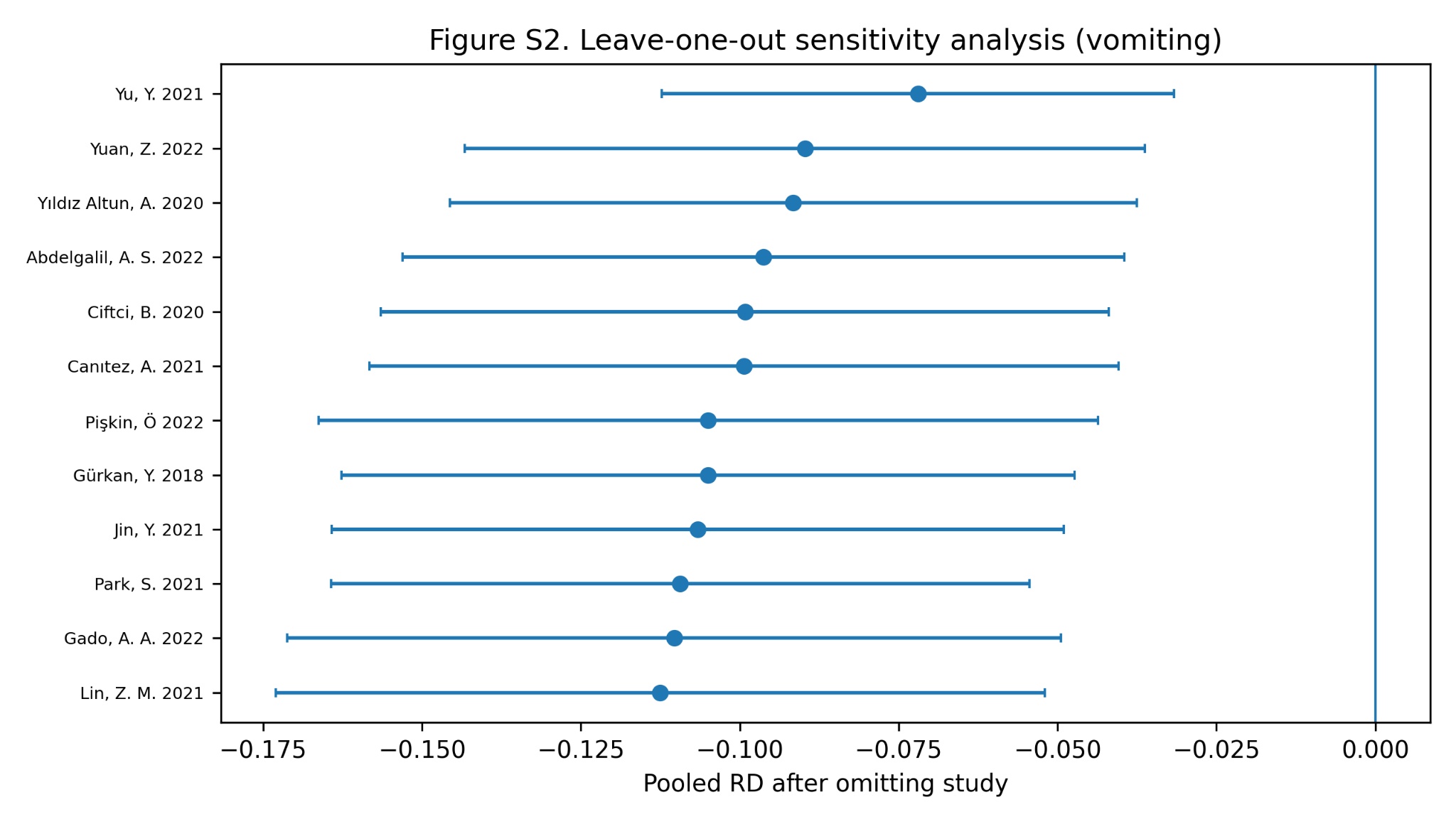

Supplement: Supplementary file 2 [file Image_2.jpg]

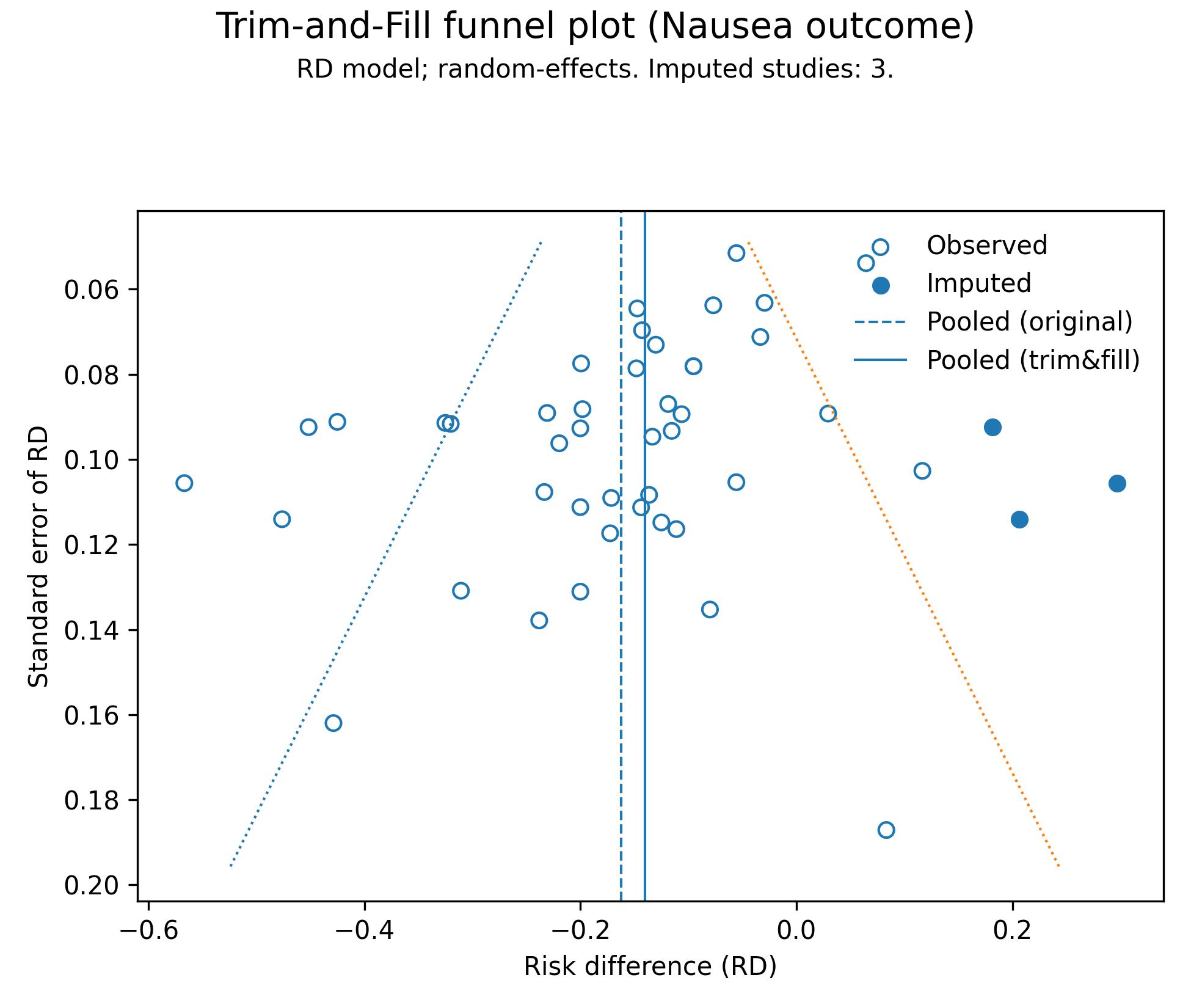

Supplement: Supplementary file 3 [file Image_3.jpg]

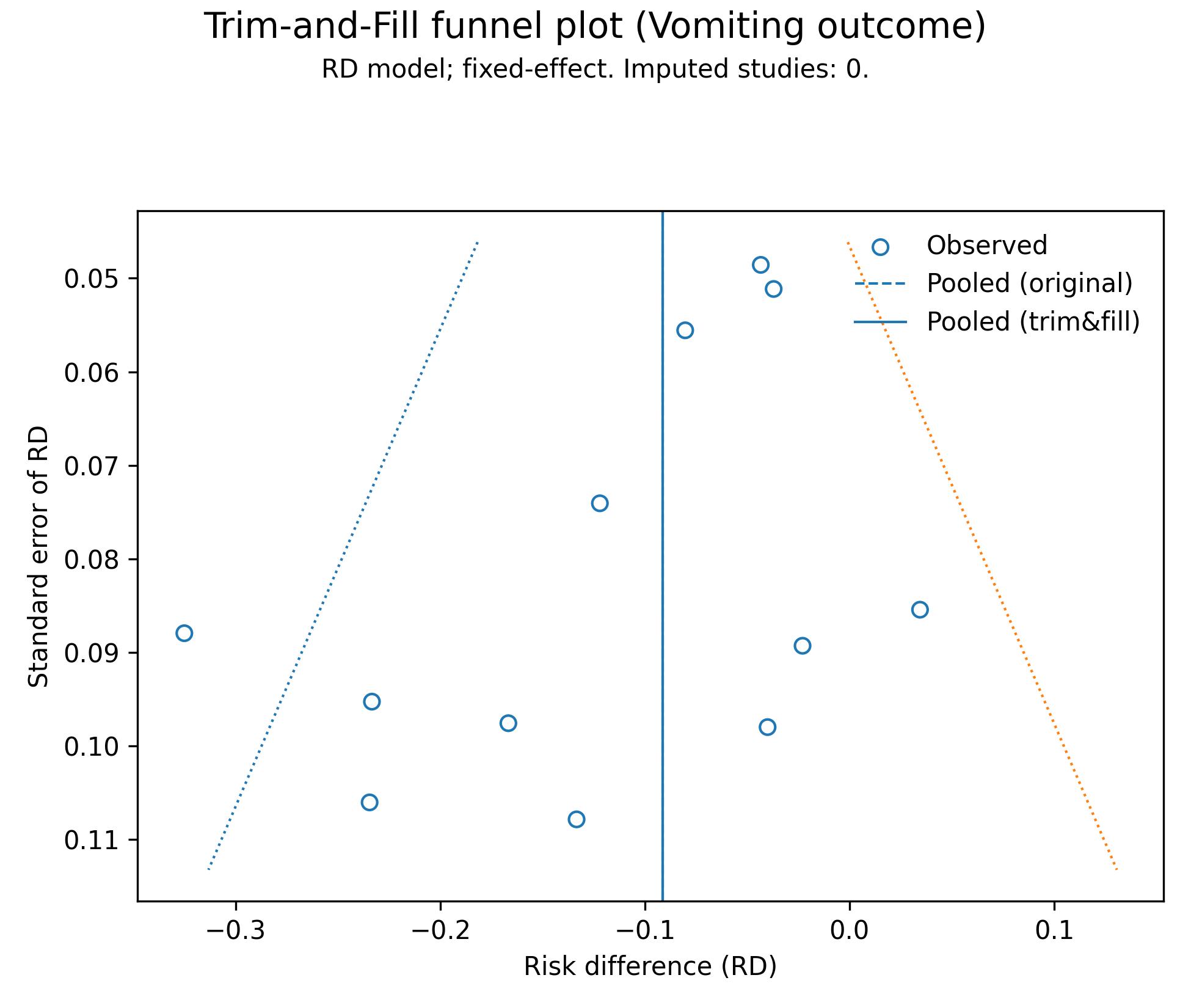

Supplement: Supplementary file 4 [file Image_4.jpg]
