# Supplementary material for: Erector spinae plane block reduces postoperative nausea and vomiting: a systematic review and meta-analysis of 44 randomized trials
Source: Front Med (Lausanne). 2026 Jan 16;12:1749998. doi: 10.3389/fmed.2025.1749998 (PMC12855405; doi:10.3389/fmed.2025.1749998)

## Supplementary Table S1. Meta-regression (univariable moderators) for nausea

| Moderator | k | Coefficient (beta) | SE | P value |
| --- | --- | --- | --- | --- |
| Age_mid | 29 | 0.0045 | 0.0027 | 0.1079 |
| prop_female | 34 | 0.0466 | 0.1350 | 0.7321 |
| BMI_mean | 32 | 0.0078 | 0.0068 | 0.2604 |
| is_TIVA | 43 | -0.0370 | 0.0497 | 0.4607 |
| antiemetic_prophylaxis | 43 | -0.0667 | 0.0436 | 0.1332 |

## Supplementary Table S2. Multivariable meta-regression for nausea

| Model | k | beta_TIVA | p_TIVA | beta_antiemetic | p_antiemetic | R^2 (WLS) |
| --- | --- | --- | --- | --- | --- | --- |
| TIVA + baseline antiemetic prophylaxis | 43 | -0.0529 | 0.2908 | -0.0757 | 0.0952 | 0.0805 |

## Supplementary Table S3. Meta-regression (univariable moderators) for vomiting

| Moderator | k | Coefficient (beta) | SE | P value |
| --- | --- | --- | --- | --- |
| Age_mid | 8 | -0.0010 | 0.0019 | 0.6341 |
| prop_female | 8 | -0.6371 | 0.6827 | 0.3867 |
| BMI_mean | 9 | 0.0111 | 0.0122 | 0.3944 |
| is_TIVA | 12 | 0.0111 | 0.0896 | 0.9040 |
| antiemetic_prophylaxis | 12 | 0.0238 | 0.0593 | 0.6962 |

## Supplementary Table S4. Leave-one-out sensitivity analysis (nausea)

| Omitted study | Pooled RD | 95% CI | I2 (%) |
| --- | --- | --- | --- |
| Abdelgalil, A. S. 2022 | -0.163 | -0.207 to -0.119 | 62.8 |
| Abu Elyazed, M. M. 2019 | -0.168 | -0.212 to -0.123 | 62.2 |
| Avis, G. 2022 | -0.167 | -0.211 to -0.122 | 62.5 |
| Bryniarski, P. 2021 | -0.168 | -0.212 to -0.125 | 61.7 |
| Canıtez, A. 2021 | -0.162 | -0.206 to -0.118 | 62.7 |
| Chiraya, S. 2023 | -0.164 | -0.208 to -0.120 | 62.9 |
| Ciftci, B. 2020 | -0.153 | -0.194 to -0.113 | 56.3 |
| Domagalska, M. 2024 | -0.155 | -0.196 to -0.113 | 57.8 |
| Dubilet, M. 2023 | -0.160 | -0.203 to -0.116 | 61.5 |
| Elshafie, M. A. 2022 | -0.156 | -0.198 to -0.114 | 59.0 |
| Fu, J. 2020 | -0.163 | -0.207 to -0.119 | 62.8 |
| Gişi, G. 2023 | -0.163 | -0.206 to -0.119 | 62.7 |
| Gökduman, H. C. 2024 | -0.164 | -0.209 to -0.120 | 62.9 |
| Gürkan, Y. 2018 | -0.165 | -0.209 to -0.121 | 62.9 |
| Hacıbeyoğlu, G. 2022 | -0.162 | -0.206 to -0.118 | 62.4 |
| Hamdi, A. A. 2023 | -0.164 | -0.208 to -0.120 | 62.9 |
| Hoogma, D. F. 2023 | -0.165 | -0.209 to -0.121 | 62.9 |
| Hu, J. 2022 | -0.165 | -0.209 to -0.120 | 62.9 |
| Jeong, H. 2022 | -0.169 | -0.212 to -0.126 | 60.7 |
| Jin, Y. 2021 | -0.164 | -0.208 to -0.120 | 62.9 |
| Lin, H. 2022 | -0.163 | -0.207 to -0.119 | 62.8 |
| Lin, Z. M. 2021 | -0.165 | -0.209 to -0.121 | 62.9 |
| Mohamed, R. M. 2023 | -0.170 | -0.211 to -0.130 | 53.8 |
| Mohasseb, A. M. 2024 | -0.166 | -0.210 to -0.122 | 62.7 |
| Park, S. 2021 | -0.164 | -0.208 to -0.120 | 62.9 |
| Peng, J. 2023 | -0.165 | -0.209 to -0.121 | 62.9 |
| Pişkin, Ö 2022 | -0.156 | -0.199 to -0.114 | 59.2 |
| Sharipova, V. 2022 | -0.160 | -0.203 to -0.117 | 61.8 |
| Sifaki, F. 2023 | -0.166 | -0.210 to -0.123 | 62.4 |
| Singh, S. 2020 | -0.166 | -0.210 to -0.121 | 62.8 |
| Soni, S. 2024 | -0.165 | -0.209 to -0.121 | 62.9 |
| Tulgar, S 2018 | -0.163 | -0.207 to -0.119 | 62.8 |
| Wang, J. 2022 | -0.165 | -0.209 to -0.121 | 62.9 |
| Wang, T. 2024 | -0.168 | -0.212 to -0.123 | 62.0 |
| Yao, Y. 2019 | -0.164 | -0.209 to -0.120 | 62.9 |
| Yao, Y. 2020 | -0.165 | -0.209 to -0.120 | 62.9 |
| Yu, Y. 2021 | -0.159 | -0.203 to -0.116 | 61.4 |
| Yuan, Z. 2022 | -0.162 | -0.206 to -0.118 | 62.6 |
| Yıldız Altun, A. 2020 | -0.161 | -0.205 to -0.118 | 62.3 |
| Zhang, J. 2023 | -0.163 | -0.207 to -0.119 | 62.7 |
| Zhu, M. 2024 | -0.165 | -0.209 to -0.120 | 62.9 |
| Zimmerer, A. 2022 | -0.168 | -0.212 to -0.124 | 61.9 |
| van den Broek, R. J. C. 2021 | -0.166 | -0.210 to -0.121 | 62.8 |

**Supplementary Table S5. Leave-one-out sensitivity analysis (vomiting)**

| Omitted study | Pooled RD | 95% CI | I2 (%) |
| --- | --- | --- | --- |
| Abdelgalil, A. S. 2022 | -0.096 | -0.153 to -0.039 | 41.8 |
| Canıtez, A. 2021 | -0.099 | -0.158 to -0.040 | 43.4 |
| Ciftci, B. 2020 | -0.099 | -0.156 to -0.042 | 43.6 |
| Gado, A. A. 2022 | -0.110 | -0.171 to -0.049 | 41.4 |
| Gürkan, Y. 2018 | -0.105 | -0.163 to -0.047 | 43.5 |
| Jin, Y. 2021 | -0.107 | -0.164 to -0.049 | 42.6 |
| Lin, Z. M. 2021 | -0.113 | -0.173 to -0.052 | 36.9 |
| Park, S. 2021 | -0.109 | -0.164 to -0.054 | 37.0 |
| Pişkin, Ö 2022 | -0.105 | -0.166 to -0.044 | 44.2 |
| Yu, Y. 2021 | -0.072 | -0.112 to -0.032 | 0.6 |
| Yuan, Z. 2022 | -0.090 | -0.143 to -0.036 | 35.0 |
| Yıldız Altun, A. 2020 | -0.092 | -0.146 to -0.038 | 36.9 |

Bubble plot of study effect size (RD) versus publication year for nausea (0–24 h). Marker size is proportional to inverse-variance weight.


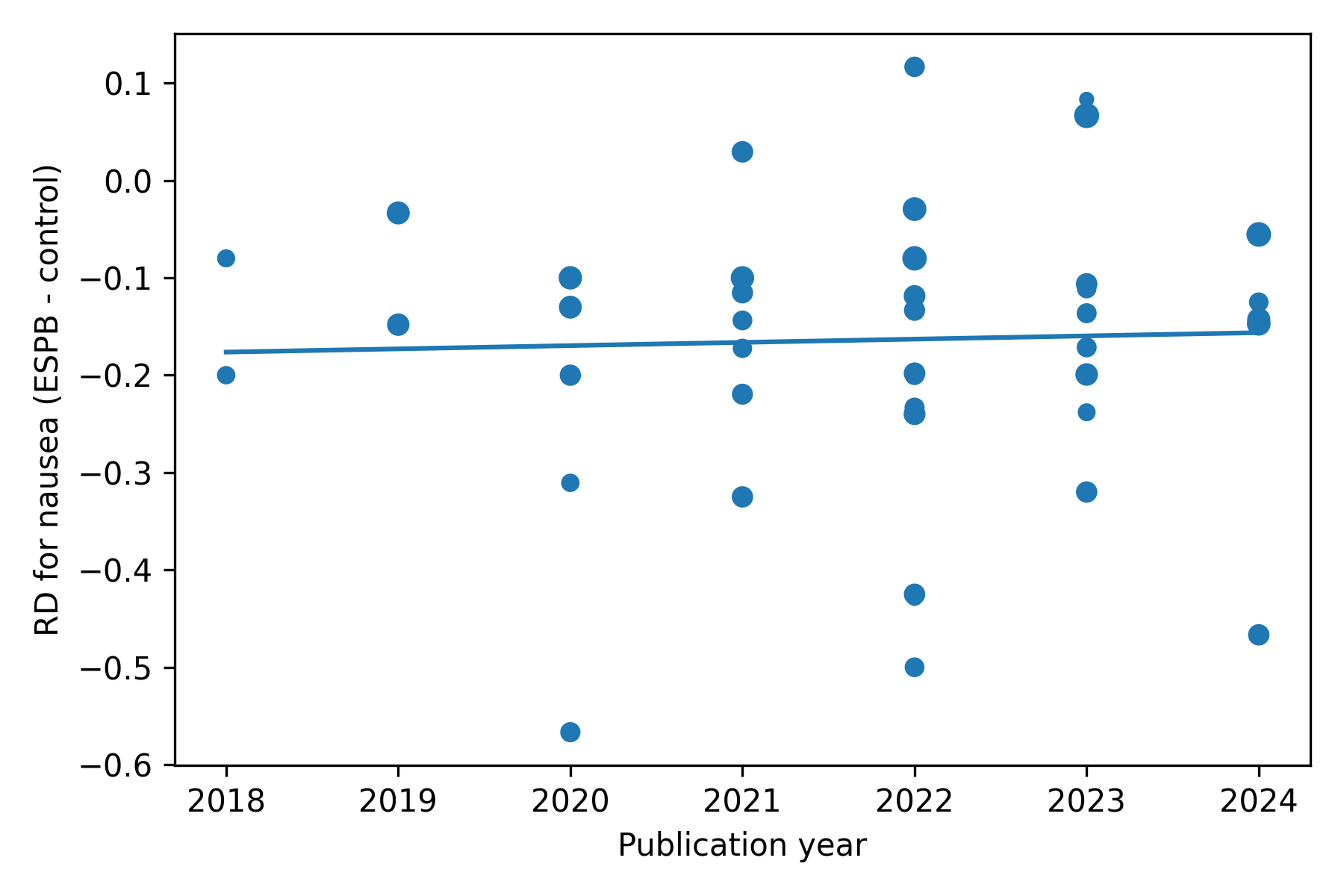

Supplement: Supplementary file 5 [file Table_1.docx]
