# Supplementary material for: Erector spinae plane block reduces postoperative nausea and vomiting: a systematic review and meta-analysis of 44 randomized trials
Source: Front Med (Lausanne). 2026 Jan 16;12:1749998. doi: 10.3389/fmed.2025.1749998 (PMC12855405; doi:10.3389/fmed.2025.1749998)
Supplement: Supplementary file 6 [file Table_2.docx]

**Supplementary Table S6: Summary of Postoperative Nausea and Vomiting (PONV) Assessment Indicators in 44 Erector Spinae Plane Block (ESPB)-Related Studies**

| Study (Author & Year) | PONV Definition | Assessment Tool | Time Point(s) |
| --- | --- | --- | --- |
| Abdelgalil et al. (2022) | Postoperative nausea and vomiting (no special modified definition) | Yes/no (frequency and percentage recording) | Postoperative 48 hours (during the entire follow-up period of adverse effects) |
| Abu Elyazed et al. (2019) | Postoperative nausea and vomiting (no special modified definition) | Yes/no (frequency recording) | Postoperative 24 hours (during the observation period of intraoperative and postoperative complications) |
| Avis et al. (2022) | Postoperative nausea and vomiting (recorded as separate "nausea" and "vomiting" events) | Yes/no (binary recording) | 1. Post-anesthesia care unit (PACU) |
|  |  |  | 2. Postoperative Day 1 |
|  |  |  | 3. Postoperative Day 2 |
|  |  |  | 4. Entire study period (up to postoperative Day 5) |
| Bryniarski et al. (2021) | Postoperative nausea and vomiting (no special modified definition) | Yes/no (frequency and percentage recording) | Postoperative 24 hours (during the observation period of postoperative adverse effects) |
| Canıtez et al. (2021) | Postoperative nausea and vomiting (no special modified definition) | 1. Nausea: 4-point scale (0=none, 1=mild, 2=moderate, 3=severe) | Postoperative 24 hours, including specific time points: T1 (postoperative onset), T2-T4 (PACU 20/40/60 min), T5-T8 (2/6/12/24 h postoperatively) |
|  |  | 2. Vomiting: Yes/no (frequency and percentage recording) |  |
| Chiraya et al. (2023) | Postoperative nausea and vomiting (no special modified definition) | Yes/no (frequency recording) | Postoperative 24 hours (during the observation period of adverse effects) |
| Ciftci et al. (2019) | Postoperative nausea and vomiting (no special modified definition) | Yes/no (frequency recording) | Postoperative 24 hours (during the observation period of opioid-related adverse events) |
| Domagalska et al. (2024) | Postoperative nausea and vomiting (no special modified definition) | Yes/no (incidence percentage recording) | Postoperative follow-up period (up to 48 hours, consistent with primary outcome assessment window) |
| Dubilet et al. (2023) | Postoperative nausea and vomiting (no special modified definition) | Yes/no (incidence percentage recording) | Postoperative 48 hours, including specific time points: PACU admission, 60 min, 4/8/12/24/48 h postoperatively |
| Elshafie et al. (2022) | Postoperative nausea and vomiting (no special modified definition) | Yes/no (frequency and percentage recording) | Postoperative 24 hours (during the observation period of postoperative adverse effects) |
| Fu et al. (2020) | Postoperative nausea and vomiting (no special modified definition) | Yes/no (incidence percentage recording) | Postoperative 48 hours (during the observation period of postoperative side effects) |
| Gado et al. (2022) | Postoperative nausea and vomiting (no special modified definition) | Yes/no (frequency recording) | Postoperative 24 hours (during the observation period of postoperative complications, synchronized with FLACC score assessment) |
| Gişi et al. (2023) | Postoperative nausea and vomiting (no special modified definition) | Yes/no (frequency recording) | Postoperative 24 hours, including specific time points: 30 min, 1/2/4/6/12/24 h postoperatively |
| Gökduman et al. (2024) | Postoperative nausea and vomiting (no special modified definition; nausea and vomiting recorded separately) | 1. Nausea: Yes/no (frequency recording) | Postoperative 60 minutes (PACU T60) and during PACU follow-up period |
|  |  | 2. Vomiting: Yes/no (frequency recording) |  |
| Gürkan et al. (2018) | Postoperative nausea and vomiting (opioid-related side effect, no special modified definition) | Yes/no (frequency recording) | Postoperative 24 hours (during the observation period of postoperative adverse effects) |
| Hacıbeyoğlu et al. (2022) | Postoperative nausea and vomiting (no special modified definition) | 1. Nausea: 4-point scale (0=none, 1=mild, 2=moderate, 3=severe) | Postoperative 24 hours (synchronized with pain score and opioid consumption assessment) |
|  |  | 2. Vomiting: Yes/no (frequency and percentage recording) |  |
| Hamdi et al. (2023) | Postoperative nausea and vomiting (no special modified definition) | Yes/no (frequency recording) | Postoperative 24 hours, including specific time points: PACU admission, 2/6/10/14/18/24 h postoperatively |
| Hoogma et al. (2023) | Postoperative nausea and vomiting (no special modified definition; classified as adverse event) | Yes/no (frequency recording) | Postoperative 24 hours |
|  |  |  | Postoperative 30 days (long-term adverse event monitoring) |
| Hu et al. (2022) | Postoperative nausea and vomiting (no special modified definition) | Yes/no (incidence percentage recording) | Postoperative 24 hours (during the observation period of postoperative side effects) |
| Jeong et al. (2022) | Postoperative nausea and vomiting (no special modified definition) | Yes/no (frequency recording) | Postoperative 24 hours (synchronized with pain score and PCA usage assessment) |
| Jin et al. (2021) | Postoperative nausea and vomiting (no special modified definition; classified as opioid-related adverse event) | Yes/no (frequency recording) | Postoperative 48 hours, including specific time points: 1/3/6/12/24/48 h postoperatively |
| Lin et al. (2022) | Postoperative nausea and vomiting (no special modified definition) | Yes/no (frequency and percentage recording) | Postoperative 48 hours, including specific time points: 0.5/1/2/4/8/24/48 h postoperatively |
| Lin et al. (2021) | Postoperative nausea and vomiting (no special modified definition; classified as treatment-related complication) | Yes/no (frequency recording) | Postoperative 12 weeks (during follow-up for adverse events) |
| Mohamed et al. (2023) | Postoperative nausea and vomiting (no special modified definition) | Yes/no (frequency recording) | Postoperative 24 hours (during the observation period of postoperative complications) |
| Mohasseb et al. (2024) | Postoperative nausea and vomiting (no special modified definition) | 4-point categorical scale (0=none, 1=mild, 2=moderate, 3=severe) | 1. Postoperative 30 minutes |
|  |  |  | 2. Postoperative 1 hour |
|  |  |  | 3. Postoperative 24 hours |
| Park et al. (2021) | Postoperative nausea and vomiting (no special modified definition) | Yes/no (frequency recording) | Postoperative 24 hours, including specific time points: 3/6/9/24 h postoperatively |
| Peng et al. (2023) | Postoperative nausea and vomiting (no special modified definition; classified as postoperative adverse reaction) | Yes/no (frequency recording) | Postoperative 24 hours and 48 hours (during follow-up for adverse reactions) |
| Pişkin et al. (2021) | Postoperative nausea and vomiting (opioid-related side effect) | Yes/no (frequency recording) | Postoperative 48 hours, including specific time points: 0/1/4/8/12/24/36/48 h postoperatively |
| Sharipova et al. (2022) | Postoperative nausea and vomiting (no special modified definition) | Yes/no (frequency and percentage recording) | Postoperative first day (within 24 hours) |
| Sifaki et al. (2023) | Postoperative nausea and vomiting (no special modified definition) | Yes/no (frequency recording) | Postoperative 96 hours, including specific time points: 12/24/48/60/72/84/96 h postoperatively |
| Singh et al. (2019) | Postoperative nausea and vomiting (no special modified definition; classified as adverse event) | Yes/no (frequency recording) | Postoperative 24 hours (during observation of postoperative adverse events) |
| Soni et al. (2024) | Postoperative nausea and vomiting (graded by severity) | 4-point scale (0=no nausea, 1=mild nausea, 2=severe nausea, 4=vomiting) | Postoperative 24 hours, including specific time points: 0/0.5/2/4/6/8/12/24 h postoperatively |
| Tulgar et al. (2018) | Postoperative nausea and vomiting (no special modified definition) | 4-point scale (none/mild/moderate/severe) for nausea; Yes/no for vomiting (frequency recording) | Postoperative 24 hours (during follow-up for adverse reactions) |
| van den Broek et al. (2021) | Postoperative nausea and vomiting (no special modified definition) | Yes/no (frequency recording) | 1. Post-anesthesia care unit (PACU) period |
|  |  |  | 2. Postoperative 24 hours (during adverse event monitoring) |
| Wang et al. (2022) | Postoperative nausea and vomiting (no special modified definition) | Verbal Rating Scale (VRS): 0=none, 1=mild nausea, 2=moderate nausea with dry-retching, 3=severe nausea with vomiting | Postoperative 3 days, including specific time points: POD1-POD3 |
| Wang et al. (2024) | Postoperative nausea and vomiting (no special modified definition) | Yes/no (frequency recording) | Postoperative 48 hours (during follow-up for opioid-related adverse events) |
| Yao et al. (2020) | Postoperative nausea and vomiting (no special modified definition) | Yes/no (frequency and percentage recording) | Postoperative 48 hours, including specific time points: 24 h and 48 h postoperatively |
| Yao et al. (2019) | Postoperative nausea and vomiting (no special modified definition) | Yes/no (frequency and percentage recording) | Postoperative 24 hours (during follow-up for adverse reactions) |
| Yıldız Altun et al. (2020) | Postoperative nausea and vomiting (no special modified definition; classified as adverse event) | Yes/no (frequency recording, categorized as none/nausea/vomiting) | Postoperative 24 hours (during observation of postoperative adverse events) |
| Yu et al. (2021) | Postoperative nausea and vomiting (no special modified definition) | Yes/no (frequency recording, nausea and vomiting separately counted) | Postoperative 24-48 hours (during adverse event monitoring) |
| Yuan et al. (2022) | Postoperative nausea and vomiting (no special modified definition) | Yes/no (frequency recording) | Postoperative 24 hours (during follow-up for opioid-related adverse events) |
| Zhang et al. (2023) | Postoperative nausea and vomiting (no special modified definition) | Yes/no (frequency and percentage recording) | Postoperative 24 hours (during acute adverse reaction monitoring) |
| Zhu et al. (2024) | Postoperative nausea and vomiting (no special modified definition) | Yes/no (frequency recording) | Postoperative 24 hours (during postoperative complication follow-up) |
| Zimmerer et al. (2022) | Postoperative nausea and vomiting (no special modified definition) | Yes/no (frequency recording) | Postoperative 24 hours (during perioperative adverse event monitoring) |
