# Supplementary material for: Erector spinae plane block reduces postoperative nausea and vomiting: a systematic review and meta-analysis of 44 randomized trials
Source: Front Med (Lausanne). 2026 Jan 16;12:1749998. doi: 10.3389/fmed.2025.1749998 (PMC12855405; doi:10.3389/fmed.2025.1749998)
Supplement: Supplementary file 7 [file Table_3.docx]

**Supplementary Table S7. Representative examples of excluded studies with reasons**

Reason **for exclusion**: The control group received other types of nerve block(n=89)

| First author | Year | Surgery/setting | ESPB comparator (control group) | Subtype of “other nerve block” in control |
| --- | --- | --- | --- | --- |
| El Halim | 2022 | Pediatric hip surgery | ESPB vs Fascia iliaca compartment block | Lower-limb plexus block |
| El Ghamry | 2019 | Modified radical mastectomy | ESPB vs Thoracic paravertebral block (TPVB/PVB) | Paravertebral block |
| Ekinci | 2020 | VATS (thoracoscopic surgery) | ESPB vs Serratus anterior plane block (SAPB) | Chest wall plane block |
| Durey | 2023 | VATS/RATS lung cancer surgery | ESPB vs Paravertebral block (PVB) | Paravertebral block |
| Duran | 2024 | Thoracotomy | ESPB vs Paravertebral block (PVB) | Paravertebral block |
| Dilsiz | 2024 | Lumbar spine surgery | ESPB vs TLIP block | Interfascial plane block |
| Chen A | 2021 | Revision total hip arthroplasty | Continuous lumbar ESPB vs Continuous lumbar plexus block (LPB) | Lumbar plexus block |
| Chen H | 2024 | Laparoscopic cholecystectomy | ESPB vs TAP block | Abdominal wall plane block |
| Zhao | 2022 | Brazilian Journal of Anesthesiology | ESPB vs Retrolaminar block (RLB) | Retrolaminar block |
| Zhang | 2024 | Journal of Clinical Anesthesia | ESPB vs Quadratus lumborum block (QLB) | Quadratus lumborum block |
| Zengin | 2022 | Journal of Cardiothoracic and Vascular Anesthesia | ESPB vs combined serratus anterior plane block (C-SAPB) | Serratus anterior plane block (deep + superficial) |
| Warner | 2022 | Pain Management | ESPB vs Transversus abdominis plane block (TAP) | Transversus abdominis plane block |

**Reason for exclusion:**
**Comparisons were based on different local anesthetic regimens within ESPB (type, concentration, or adjuvants)(n=77)**

| First author | Year | Surgery | Comparison | Subtype (specific) |
| --- | --- | --- | --- | --- |
| Wang X | 2021 | Mastectomy | ESPB + ropivacaine vs ESPB + ropivacaine + dexmedetomidine | Adjuvant (dexmedetomidine) vs no adjuvant |
| Wang Q | 2022 | Thoracotomy | ESPB + ropivacaine vs ESPB + ropivacaine + dexmedetomidine | α2-agonist adjuvant comparison |
| ul Huda A | 2024 | Lumbar spine | ESPB + ropivacaine vs ESPB + ropivacaine + dexmedetomidine | Adjuvant (dexmedetomidine) |
| Sifaki F | 2022 | Laparoscopic cholecystectomy | ESPB + saline vs ESPB + ropivacaine ± dexmedetomidine | Adjuvant vs placebo within ESPB |
| Sherif F | 2022 | Mastectomy | ESPB + levobupivacaine vs + ketamine vs + magnesium | Different pharmacologic adjuvants |
| Refaat S | 2023 | Spine surgery | ESPB + bupivacaine + MgSO₄ vs + dexmedetomidine | Adjuvant head-to-head comparison |
| Reddy S | 2024 | PCNL | ESPB + bupivacaine + dexmedetomidine vs fentanyl | Different opioid/α2 adjuvants |
| Rao J | 2021 | VATS | ESPB + ropivacaine + nalbuphine vs + dexmedetomidine | Opioid vs α2-agonist adjuvant |
| Nousheen S | 2024 | Laparoscopic surgery | ESPB + bupivacaine vs + dexamethasone | Steroid adjuvant |
| Mathew N | 2024 | Laparoscopic cholecystectomy | ESPB + dexmedetomidine vs fentanyl | Adjuvant comparison (observational) |
| Gao Z | 2019 | VATS | ESPB + dexmedetomidine vs + dexamethasone vs none | Multiple adjuvants within ESPB |
| Gao X | 2021 | VATS | ESPB + different doses of dexmedetomidine | Dose–response of adjuvant |
| Elshal M | 2021 | Thoracotomy | ESPB + bupivacaine vs + dexmedetomidine | Adjuvant comparison |
| Akshay S | 2023 | Thoracic surgery | ESPB + ropivacaine vs + dexmedetomidine | Adjuvant comparison |
| Ahmed H | 2023 | Mastectomy | ESPB + levobupivacaine + dexmedetomidine vs dexamethasone | Adjuvant comparison |
| Abu El Hassan | 2024 | Spine fixation | ESPB + bupivacaine vs + dexmedetomidine vs saline | Adjuvant vs placebo |
| Abdelbadie M | 2022 | Lumbar fusion | ESPB + bupivacaine vs + magnesium | Mineral adjuvant |
| Cao X | 2024 | Mastectomy | ESPB + ropivacaine vs + hydromorphone | Opioid adjuvant |

**Reason for exclusion:**
**Not an original randomized controlled trial (secondary research such as systematic review, meta-analysis, or network meta-analysis).(n=61)**

| First author | Year | Title (short) | Journal | Reference type | Specific reason |
| --- | --- | --- | --- | --- | --- |
| Zhou K | 2023 | Regional anesthetic techniques after cardiac surgery | Front Cardiovasc Med | Network meta-analysis | Secondary study; not an original RCT |
| Zeng J | 2022 | Regional analgesia for VATS | Pain Physician | Network meta-analysis | Secondary study; not an original RCT |
| Wong HY | 2021 | Anesthesia modalities in breast surgery | J Clin Anesth | Network meta-analysis | Secondary study; not an original RCT |
| Singh NP | 2022 | Regional anesthesia for oncologic breast surgery | Can J Anaesth | Network meta-analysis | Secondary study; not an original RCT |
| Wang J | 2021 | Local anesthetic techniques after cesarean section | J Pain Res | Network meta-analysis | Secondary study; not an original RCT |
| Scorsese G | 2023 | Thoracic wall blocks in VATS | J Pain Res | Network meta-analysis | Secondary study; not an original RCT |
| Zhang Y | 2021 | ESPB in breast cancer surgery | BMC Anesthesiol | Systematic review & meta-analysis | Secondary study; not an original RCT |
| Yu L | 2023 | Dexmedetomidine as ESPB adjuvant | BMC Anesthesiol | Systematic review & meta-analysis | Secondary study; not an original RCT |
| Yang X | 2023 | ESPB in laparoscopic cholecystectomy | BMC Anesthesiol | Systematic review & meta-analysis | Secondary study; not an original RCT |
| Xiong C | 2021 | PVB vs ESPB | PLoS One | Meta-analysis | Secondary study; not an original RCT |
| Wu S | 2024 | Bilateral ESPB in spine surgery | World Neurosurg | Meta-analysis | Secondary study; not an original RCT |
| Wilson AA | 2024 | ESPB in lumbar surgery | J Perianesth Nurs | Meta-analysis | Secondary study; not an original RCT |
| Weng WT | 2021 | ESPB vs PVB in breast surgery | Pain Physician | Meta-analysis | Secondary study; not an original RCT |
| Viderman D | 2022 | ESPB in thoracolumbar surgery | Front Med | Meta-analysis | Secondary study; not an original RCT |
| Viderman D | 2022 | ESPB in abdominal surgery | Front Med | Meta-analysis | Secondary study; not an original RCT |
| Sun Q | 2023 | ESPB in lumbar surgery | BMC Anesthesiol | Meta-analysis | Secondary study; not an original RCT |
| Stewart M | 2023 | ESPB vs TAP block | AANA J | Meta-analysis | Secondary study; not an original RCT |
| Singh NP | 2020 | ESPB in oncologic breast surgery | Breast J | Meta-analysis & TSA | Secondary study; not an original RCT |
| Wang J | 2023 | QLB in nephrectomy | Pain Med | Meta-analysis | Secondary study; not an original RCT |

**Table footnote：**

**This table presents representative examples of records excluded after full-text review. Due to space constraints, not all excluded records (n = 227) are listed individually. All records were screened against the prespecified eligibility criteria (PICOS framework) and categorized according to the primary reason for exclusion. The complete exclusion log is available from the authors upon reasonable request.**
