# Supplementary material for: Erector spinae plane block reduces postoperative nausea and vomiting: a systematic review and meta-analysis of 44 randomized trials
Source: Front Med (Lausanne). 2026 Jan 16;12:1749998. doi: 10.3389/fmed.2025.1749998 (PMC12855405; doi:10.3389/fmed.2025.1749998)
Supplement: Supplementary file 8 [file Table_4.docx]

Baseline body mass index (BMI) and perioperative anti-emetic prophylaxis strategies in included randomized controlled trials

| Author(Year) | Anti-emetic Prophylaxis | BMI |
| --- | --- | --- |
| Abdelgalil et al. (2022) | No (Ondansetron 0.1 mg/kg IV was administered as rescue only for moderate to severe PONV postoperatively) | Reported, ESPB Group: 27.9±4.38 kg/m²; Group C: 28.8±4.00 kg/m² |
| Abu Elyazed et al. (2019) | No (No routine preoperative/intraoperative anti-emetic prophylaxis; only PONV incidence was recorded) | Not reported (Only weight data provided, no BMI calculation) |
| Avis et al. (2022) | Yes (Dexamethasone 8 mg IV after induction; Ondansetron 8 mg or Droperidol 1.25 mg IV as needed postoperatively) | Reported, Saline group: 26(24–31) kg/m²; ESPB Group: 28(24–32) kg/m² (Median and IQR) |
| Bryniarski et al. (2021) | No (Only PONV incidence was recorded; no routine preoperative/intraoperative anti-emetic prophylaxis) | Reported, ESPB Group: 28.5±3.6 kg/m²; Control Group: 28.7±3.7 kg/m² |
| Canıtez et al. (2021) | No (Metoclopramide 5 mg IV was administered as rescue only for PONV postoperatively; no routine prophylaxis) | Reported, Group N: 26.2±3.5 kg/m²; ESPB Group: 27.0±2.4 kg/m² |
| Chiraya et al. (2023) | No (Only PONV incidence was recorded; no routine preoperative/intraoperative anti-emetic prophylaxis) | Reported, ESPB Group: 25.44±3.07 kg/m²; Control group: 25.02±2.68 kg/m² |
| Ciftci et al. (2019) | Yes (Ondansetron 4 mg IV administered intraoperatively as routine anti-emetic prophylaxis) | Not reported (Only height and weight data provided, no BMI calculation) |
| Domagalska et al. (2024) | Yes (Dexamethasone 0.1 mg/kg IV administered intravenously 30 minutes preoperatively as part of multimodal analgesia) | Not reported (Only weight and height data provided, no BMI calculation) |
| Dubilet et al. (2023) | No (No routine preoperative/intraoperative anti-emetic prophylaxis; only PONV incidence was recorded) | Not reported (No BMI data provided) |
| Elshafie et al. (2022) | No (Only PONV incidence was recorded; no routine preoperative/intraoperative anti-emetic prophylaxis) | Reported, ESPB Group: 30.00(28.00–33.00) kg/m²; Conventional Group: 31.00(22.00–36.00) kg/m² (Median and IQR) |
| Fu et al. (2020) | No (Metoclopramide 10 mg IV was administered as rescue only for PONV postoperatively; no routine prophylaxis) | Reported, ESPB Group: 21.4±1.5 kg/m²; Group B: 22.0±1.9 kg/m² |
| Gado et al. (2022) | Yes (Dexamethasone 0.1 mg/kg IV + Granisetron 10 μg/kg IV administered intraoperatively for PONV prevention) | Not reported (Only weight and height data provided, no BMI calculation) |
| Gişi et al. (2023) | No (No routine preoperative/intraoperative anti-emetic prophylaxis; only PONV incidence was recorded) | Not reported (Only weight and height data provided, no BMI calculation) |
| Gökduman et al. (2024) | No (No routine preoperative/intraoperative anti-emetic prophylaxis; dexamethasone used as analgesic adjuvant; PONV managed postoperatively) | Reported, ESPB Group: 26.4±3.9 kg/m²; IV Group: 26.3±4.2 kg/m² |
| Gürkan et al. (2018) | Yes (Ondansetron 8 mg IV administered intraoperatively as routine anti-emetic prophylaxis) | Reported, ESPB Group: 27.88±4.66 kg/m²; Control Group: 28.29±4.43 kg/m² |
| Hacıbeyoğlu et al. (2022) | Yes (Ondansetron 0.1 mg/kg IV administered intraoperatively as routine anti-emetic prophylaxis) | Reported, Control Group: 26.6±1.9 kg/m²; ESPB Group: 25.8±2.5 kg/m² |
| Hamdi et al. (2023) | Yes (Ondansetron 4 mg IV administered preoperatively as routine anti-emetic prophylaxis) | Reported, Group C: 25.3±2.3 kg/m²; ESPB Group: 24.8±1.9 kg/m² |
| Hoogma et al. (2023) | Yes (Dexamethasone 5 mg IV + Ondansetron 4 mg IV administered intraoperatively for PONV prevention) | Reported, ESPB Group: 26±3.5 kg/m²; Control Group: 25±3.6 kg/m² |
| Hu et al. (2022) | No (No routine preoperative/intraoperative anti-emetic prophylaxis; dexamethasone included in postoperative PCIA) | Not reported (No specific BMI data provided) |
| Jeong et al. (2022) | No (No routine preoperative/intraoperative anti-emetic prophylaxis; PONV managed with ramosetron hydrochloride 0.3 mg IV postoperatively) | Reported, Control Group: 23.8±3.0 kg/m²; ESPB Group: 24.5±4.0 kg/m² |
| Jin et al. (2021) | No (No routine preoperative/intraoperative anti-emetic prophylaxis; palonosetron used in postoperative PCIA for rescue) | Reported, Group G: 24.311±4.861 kg/m²; Group ESPB: 23.882±5.270 kg/m² |
| Lin et al. (2022) | Yes (Dexamethasone 10 mg IV + Tropisetron 5 mg IV administered intraoperatively for PONV prevention) | Reported, ESPB Group: 23.4[21.9–26.5] kg/m²; Control Group: 24.2[22.5–25.5] kg/m² (Median and IQR) |
| Lin et al. (2021) | No (No routine preoperative/intraoperative anti-emetic prophylaxis; PONV managed with postoperative medications) | Reported, Control Group: 24.0±1.9 kg/m²; ESPB Group: 24.9±2.9 kg/m² |
| Mohamed et al. (2023) | No (No routine preoperative/intraoperative anti-emetic prophylaxis; PONV reported postoperatively in 2 patients) | Reported, Pregabalin Group: 28.98±2.04 kg/m²; ESPB Group: 29.50±2.13 kg/m² |
| Mohasseb et al. (2024) | No (No routine preoperative/intraoperative anti-emetic prophylaxis; PONV managed with metoclopramide 10 mg IV postoperatively) | Reported, ESPB Group: 35.53±6.39 kg/m²; Ket-Dex Group: 34.03±5.84 kg/m² |
| Park et al. (2021) | Yes (Palonosetron HCl 0.075 mg IV administered 30 minutes preoperatively as routine anti-emetic prophylaxis) | Reported, IV-PCA alone Group: 22.9±3.1 kg/m²; ESPB Group: 22.4±3.5 kg/m² |
| Peng et al. (2023) | Yes (Tropisetron 5 mg IV administered before surgical closure as routine anti-emetic prophylaxis) | Reported, ESPB Group: 26.4±1.8 kg/m²; Control Group: 25.7±2.2 kg/m² |
| Pişkin et al. (2021) | Yes (Ondansetron 8 mg IV administered 20 minutes pre-extubation as routine anti-emetic prophylaxis) | Not reported (Only height and weight data provided, no BMI calculation) |
| Sharipova et al. (2022) | Yes (Dexamethasone 4 mg IV after induction + Metoclopramide 30 mg IV at end of surgery as routine prophylaxis) | Not reported (No BMI data provided) |
| Sifaki et al. (2023) | No (No routine preoperative/intraoperative anti-emetic prophylaxis; PONV recorded postoperatively) | Reported, ESPB Group: 26.7±2.84 kg/m²; CL Group: 28.7±3.93 kg/m² |
| Singh et al. (2019) | No (No routine preoperative/intraoperative anti-emetic prophylaxis; PONV managed with metoclopramide postoperatively) | Reported, Control Group: 24.7±1.6 kg/m²; ESPB Group: 25.1±1.8 kg/m² |
| Soni et al. (2024) | No (No routine preoperative/intraoperative anti-emetic prophylaxis; Ondansetron 4 mg IV as rescue for PONV) | Not reported (No specific BMI data provided) |
| Tulgar et al. (2018) | No (No routine preoperative/intraoperative anti-emetic prophylaxis; Ondansetron administered as rescue for PONV) | Reported, ESPB Group (Group B): 29.9±3.7 kg/m²; Control Group (Group C): 28.9±4.9 kg/m² |
| van den Broek et al. (2021) | No (No routine preoperative/intraoperative anti-emetic prophylaxis; PONV managed postoperatively) | Reported, No block Group: 27.0±4.0 kg/m²; ESP Block Group: 26.7±4.6 kg/m² |
| Wang et al. (2022) | Yes (Dexamethasone 5 mg IV + Ramosetron 0.3 mg IV administered as routine PONV prophylaxis) | Reported, PCIA Group: 23.4±2.6 kg/m²; ESPB Group: 22.9±2.9 kg/m² |
| Wang et al. (2024) | Yes (Ondansetron 4 mg IV + Butorphanol 1 mg IV administered preoperatively as routine anti-emetic prophylaxis) | Reported, ICNB-PCIA Group: 24.0±3.76 kg/m²; ESPB Group: 22.9±3.72 kg/m² |
| Yao et al. (2020) | Yes (Dexamethasone 10 mg + Tropisetron 5 mg IV administered after tracheal intubation as routine prophylaxis) | Not reported (Only height and weight median/IQR provided, no BMI calculation) |
| Yao et al. (2019) | No (Ondansetron 4 mg + Dexamethasone 5 mg IV administered as rescue for PONV; no routine prophylaxis) | Not reported (Only height and weight mean±SD provided, no BMI calculation) |
| Yıldız Altun et al. (2020) | No (Metoclopramide IV administered as rescue for PONV; no routine prophylaxis) | Reported, ESPB Group (Group 1): 25.28±2.26 kg/m²; Control Group (Group 2): 25.53±2.73 kg/m² |
| Yu et al. (2021) | Yes (Tropisetron 5 mg IV administered before skin closure as routine PONV prophylaxis) | Reported, ESPB Group: 21±3 kg/m²; PCA Group: 22±2 kg/m² |
| Yuan et al. (2022) | No (No routine preoperative/intraoperative anti-emetic prophylaxis; PONV incidence recorded postoperatively) | Reported, ESPB Group: 16.37±0.90 kg/m²; GA Group: 16.71±1.23 kg/m² |
| Zhang et al. (2023) | Yes (Dexamethasone 10 mg + Tropisetron 5 mg IV administered preoperatively as routine PONV prophylaxis) | Reported, Group A (GA): 23.21±2.26 kg/m²; ESPB Group: 23.38±2.52 kg/m² |
| Zhu et al. (2024) | No (No routine preoperative/intraoperative anti-emetic prophylaxis; PONV incidence recorded postoperatively) | Reported, ESPB Group: 23.52±2.14 kg/m²; Control Group: 24.48±2.73 kg/m² |
| Zimmerer et al. (2022) | Yes (Dexamethasone 4 mg + Ondansetron 4 mg IV administered intraoperatively for PONV prevention) | Reported, ESPB Group: 24.6±2.2 kg/m²; Control Group: 23.1±2.5 kg/m² |
