# Supplementary material for: Erector spinae plane block reduces postoperative nausea and vomiting: a systematic review and meta-analysis of 44 randomized trials
Source: Front Med (Lausanne). 2026 Jan 16;12:1749998. doi: 10.3389/fmed.2025.1749998 (PMC12855405; doi:10.3389/fmed.2025.1749998)
Supplement: Supplementary file 9 [file Table_5.docx]

**PubMed (MEDLINE):**

( "erector spinae plane block"[Title/Abstract]

OR "erector spinae plane"[Title/Abstract]

OR "erector spinae block"[Title/Abstract]

OR "erector spinae plane blockade"[Title/Abstract]

OR ESPB[Title/Abstract])

AND( "postoperative nausea and vomiting"[Title/Abstract]

OR PONV[Title/Abstract]

OR nausea[Title/Abstract]

OR vomiting[Title/Abstract]

OR emesis[Title/Abstract]

OR "Nausea"[Mesh]

OR "Vomiting"[Mesh]

OR "Postoperative Nausea and Vomiting"[Mesh])

AND( randomized controlled trial[Publication Type]

OR controlled clinical trial[Publication Type]

OR randomi*ed[Title/Abstract]

OR placebo[Title/Abstract]

OR randomly[Title/Abstract]

OR trial[Title/Abstract])

NOT( animals[Mesh] NOT humans[Mesh])

**Embase:**

( 'erector spinae plane block':ti,ab,kw

OR 'erector spinae plane':ti,ab,kw

OR 'erector spinae block':ti,ab,kw

OR espb:ti,ab,kw)

AND( 'postoperative nausea and vomiting'/exp

OR 'nausea'/exp

OR 'vomiting'/exp

OR ponv:ti,ab,kw

OR nausea:ti,ab,kw

OR vomiting:ti,ab,kw

OR emesis:ti,ab,kw)

AND( 'randomized controlled trial'/exp

OR 'randomization'/exp

OR 'double blind procedure'/exp

OR 'single blind procedure'/exp

OR random*:ti,ab,kw

OR placebo*:ti,ab,kw)

AND[humans]/lim

**Cochrane Central Register of Controlled Trials (CENTRAL):**

(erector spinae plane block OR erector spinae plane OR erector spinae block OR ESPB)

AND(postoperative nausea and vomiting OR PONV OR nausea OR vomiting OR emesis)

In addition, the reference lists of relevant reviews and included trials were manually screened to identify any additional eligible studies.
